# Molecular phylogeny and phylogeography of the freshwater-fish genus *Pethia* (Teleostei: Cyprinidae) in Sri Lanka

**DOI:** 10.1186/s12862-021-01923-5

**Published:** 2021-11-10

**Authors:** Hiranya Sudasinghe, Tharindu Ranasinghe, Jayampathi Herath, Kumudu Wijesooriya, Rohan Pethiyagoda, Lukas Rüber, Madhava Meegaskumbura

**Affiliations:** 1 Evolutionary Ecology and Systematics Laboratory, Department of Molecular Biology and Biotechnology, University of Peradeniya, Peradeniya, 20400 Sri Lanka; 2 Postgraduate Institute of Science, University of Peradeniya, Peradeniya, 20400 Sri Lanka; 3 Evolutionary Ecology, Institute of Ecology and Evolution, University of Bern, 3012 Bern, Switzerland; 4 Naturhistorisches Museum Bern, Bernastrasse, 15, 3005 Bern, Switzerland; 5 Butterfly Conservation Society of Sri Lanka, 762/A, Yatihena Malwana, 11670 Sri Lanka; 6 Guangxi Key Laboratory for Forest Ecology and Conservation, College of Forestry, Guangxi University, Nanning, 530004 Guangxi People’s Republic of China; 7 Department of Zoology, Faculty of Science, University of Peradeniya, Peradeniya, 20400 Sri Lanka; 8 Ichthyology Section, Australian Museum, 6 College Street, Sydney, NSW 2010 Australia; 9 Aquatic Ecology and Evolution, Institute of Ecology and Evolution, University of Bern, 3012 Bern, Switzerland

**Keywords:** Smiliogastrinae, Morphology, Barb, Biodiversity hotspot, India

## Abstract

**Background:**

Sri Lanka is a continental island separated from India by the Palk Strait, a shallow-shelf sea, which was emergent during periods of lowered sea level. Its biodiversity is concentrated in its perhumid south-western ‘wet zone’. The island’s freshwater fishes are dominated by the Cyprinidae, characterized by small diversifications of species derived from dispersals from India. These include five diminutive, endemic species of *Pethia* (*P. bandula*, *P. cumingii*, *P. melanomaculata*, *P. nigrofasciata*, *P. reval*), whose evolutionary history remains poorly understood. Here, based on comprehensive geographic sampling, we explore the phylogeny, phylogeography and morphological diversity of the genus in Sri Lanka.

**Results:**

The phylogenetic analyses, based on mitochondrial and nuclear loci, recover Sri Lankan *Pethia* as polyphyletic. The reciprocal monophyly of *P. bandula* and *P. nigrofasciata*, and *P. cumingii* and *P. reval*, is not supported. *Pethia nigrofasciata*, *P. cumingii*, and *P. reval* show strong phylogeographic structure in the wet zone, compared with *P. melanomaculata*, which ranges across the dry and intermediate zones. Translocated populations of *P. nigrofasciata* and *P. reval* in the Central Hills likely originate from multiple sources. Morphological analyses reveal populations of *P. nigrofasciata* proximal to *P. bandula*, a narrow-range endemic, to have a mix of characters between the two species. Similarly, populations of *P. cumingii* in the Kalu basin possess orange fins, a state between the red-finned *P. reval* from Kelani to Deduru and yellow-finned *P. cumingii* from Bentara to Gin basins.

**Conclusions:**

Polyphyly in Sri Lankan *Pethia* suggests two or three colonizations from mainland India. Strong phylogeographic structure in *P. nigrofasciata*, *P. cumingii* and *P. reval*, compared with *P. melanomaculata*, supports a model wherein the topographically complex wet zone harbors greater genetic diversity than the topographically uniform dry-zone. Mixed morphological characters between *P. bandula* and *P. nigrofasciata*, and *P. cumingii* and *P. reval*, and their unresolved phylogenies, may suggest recent speciation scenarios with incomplete lineage sorting, or hybridization.

**Supplementary Information:**

The online version contains supplementary material available at 10.1186/s12862-021-01923-5.

## Background

As part of a global biodiversity hotspot, the 65,000 km^2^ island of Sri Lanka contains remarkable biotic endemism [1, 2]. The island is narrowly separated from India by the ~ 25 km wide Palk Strait, a shallow-shelf sea. Marine regressions in excess of 10 m have been frequent since the Oligocene [3], giving rise to a wide land bridge (the Palk Isthmus) connecting Sri Lanka and India [4, 5]. Despite Sri Lanka’s Gondwanan origins [6], its vertebrate fauna lacks a clear Gondwanan signature except in the case of a single lineage of amphibians [7]. Thus, the Palk Isthmus has been the only route for the dispersal of aquatic organisms between the mainland and Sri Lanka. Despite periodic inundations during sea-level high stands, the isthmus has been exposed for most of the past 15 My and until as recently as 10 kya [3, 8].

Endemism in the island, which is part of the Western Ghats-Sri Lanka Biodiversity Hotspot [9], is concentrated in its hilly, perhumid south-western wet zone (rainfall > 2.5 m/y), characterized by mixed-dipterocarp rainforests and complex topography [2]. By contrast, the topographically uniform dry zone (rainfall < 1.8 m/y) is markedly more seasonal, characterized by deciduous forest with relatively low endemism. Between these is a narrow ‘intermediate zone’ [2].

Despite extensive terrestrial connectivity with the mainland, however, biotic exchange across the Palk Isthmus appears to have been strongly mediated by climate [5, 8, 10]. Arid conditions on the isthmus led, especially during the Plio-Pleistocene, to it serving more as a filter than a conduit for biotic exchange [5, 8, 10, 11]. Hence, much of the island’s remarkable biodiversity derives from insular diversifications stemming from a small number of immigrant dispersals, as in the case of its 59 endemic treefrog species and 50 endemic crab species, shown to be monophyletic by Meegaskumbura et al. [12] and Beenaerts et al. [13], respectively. The same has been shown to be true also for several freshwater-fish diversifications, such as in the cypriniform genera *Devario*, *Rasbora* and *Systomus* [11, 14, 15].

The cyprinid genus *Pethia* presently includes some 43 valid species (Additional file 1: Table S1), nine of which have been discovered since the genus was first described by Pethiyagoda et al. [16] [see: 17]. *Pethia* have a wide distribution, ranging from Sri Lanka across India and on to Myanmar [16, 18–21]. They are colorful, usually sexually dimorphic fishes that have long been popular in the global ornamental-fish trade [22]. Members of the genus are united by a suite of characters (none of them exclusive) that include small size (standard length up to about 5 cm), a posteriorly serrated last unbranched dorsal-fin ray, having the lateral line (usually) incomplete, and exhibiting between one and three black blotches, bars or spots on the side of the body, usually including one in the humeral-cleithral region and another above the anal fin or on the caudal peduncle [16]. Although the phylogenetic relationships of *Pethia* to the genera formerly referred to *Puntius* sensu lato, in which the genus was subsumed prior to Pethiyagoda et al. [16], remain only weakly supported, several molecular studies have shown *Pethia* to be monophyletic [10, 11, 23–26]. Phylogenetic relationships within the genus, however, remain to be elucidated.

Five species of *Pethia* have been reported from Sri Lanka: *Pethia bandula, P. cumingii, P. nigrofasciata, P. reval* and *P. melanomaculata,* all of them endemic [27, 28]. The genus is widely distributed in the island’s major habitat types, from sea level to elevations of about 1000 m above sea level (a.s.l) [22, 27]. The first three species are endemic to the wet zone, while the last occurs in the dry and intermediate zones. *Pethia reval* ranges from the Kelani basin in the wet-zone to the Deduru basin in the intermediate zone, while *P. bandula* is a narrow-range endemic confined to a short stretch of a single wet-zone stream draining the Kelani basin [22, 27–29]. *Pethia reval* and *P. cumingii* are distinguished primarily by fin coloration: red in the former and yellow in the latter [28].

The four wet-zone species, as a group, differ from *Pethia melanomaculata* in exhibiting near-complete allopatry as well as a coloration that includes two or three black bars on the body, compared with a small black spot on the humeral region and a blotch on the caudal peduncle of *P. melanomaculata.* This and other differences in morphology [27–29] lead us to hypothesize that the Sri Lankan diversification of *Pethia* is not monophyletic. The wet-zone species appear to comprise a monophyletic diversification, while *P. melanomaculata* seems more closely related to *P. punctata*, a South Indian species [27, 30, 31]. Additionally, the wet-zone species have unusual distributions. While *P. reval* and *P. cumingii* are apparently allopatric, *P. nigrofasciata* occurs in sympatry with both; and while the range of *P. bandula* lies within the range of both *P. nigrofasciata* and *P. reval*, *P. bandula* does not occur in syntopy with either. Given the wet-zone’s more complex topography, we hypothesize that the three widespread wet-zone species (*P. reval*, *P. cumingii,* and *P. nigrofasciata*) exhibit strong phylogeographic structure in comparison to *P. melanomaculata*, which occurs across the topographically uniform dry-zone plains.

**Fig. 1:**
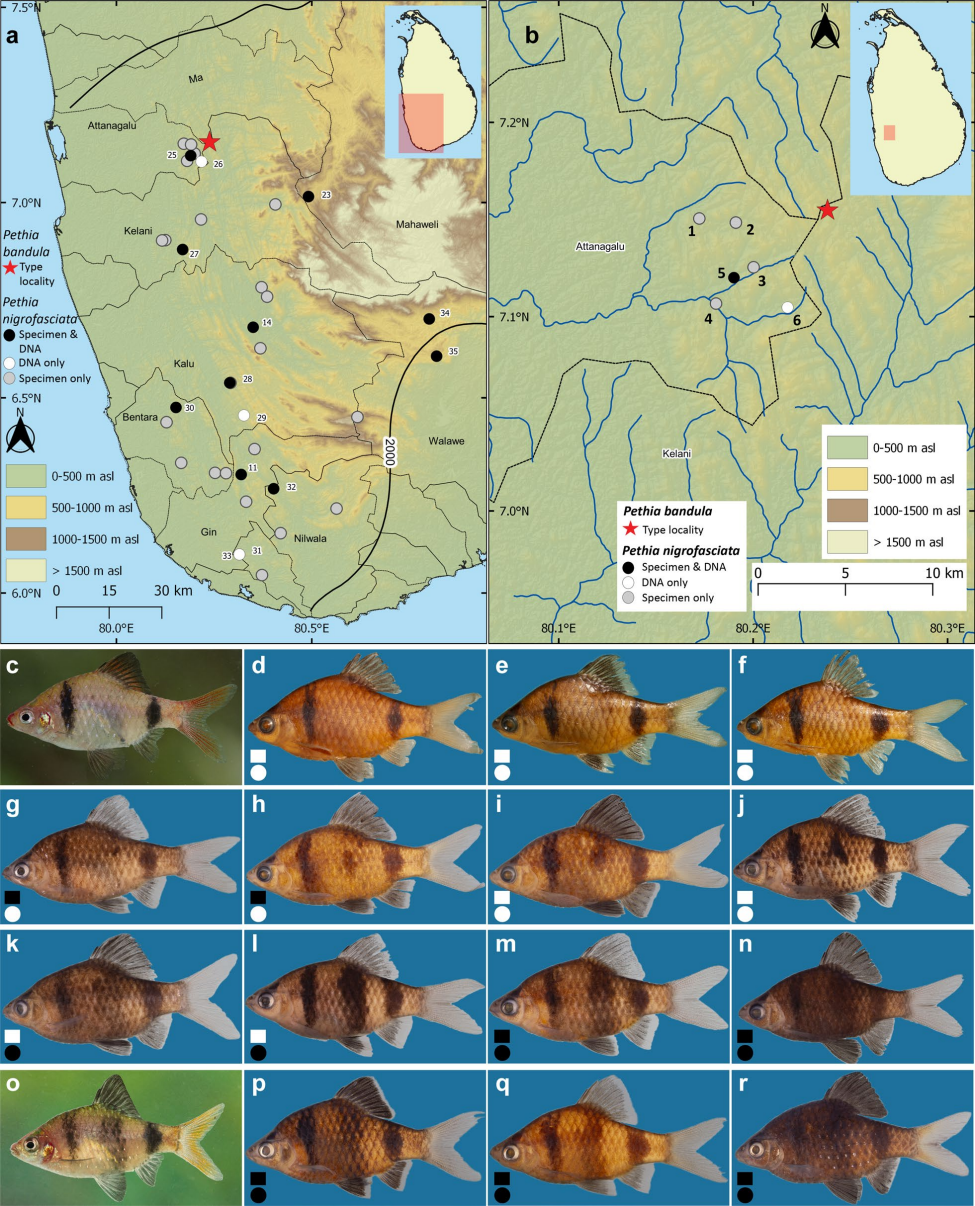
**a** Sri Lanka, showing the geographical origin of samples of *Pethia nigrofasciata* and *P. bandula* used in this study. The thin black lines indicate basin boundaries, while the bold black line indicates the 2000-mm isohyet, which encompasses the wet zone. Numbers on the map (**a**) represent the sampling localities listed in Table 1. **b** close-up of the sampling localities for *P. nigrofasciata* in the headwaters of the Attanagalu basin neighboring the type locality of *P. bandula*. These populations show a mix of meristic characters and color patterns intermediate between *P. nigrofasciata* and *P. bandula*. In **d–r**, specimens with complete and incomplete pored lateral-line scales are represented by black and white squares, respectively while those with complete bar beneath dorsal fin and those without or an incomplete are represented by black and white circles, respectively. Numbers on the map (**b**) are referenced to the specimens illustrated in (**g**–**n**) in parentheses. **c** Live coloration of *P. bandula*. **d** Holotype of *P. bandula*, ZRC 38,483, 34.6 mm SL, **e** paratype CMK 7146C, 31.6 mm SL, and **f** paratype, CMK 7146D, 30.8 mm SL of *P. bandula*. *Pethia* cf. *nigrofasciata* from the Attanagalu basin in **g**–**n**. **g** DZ5353F, 40.3 mm SL (2). **h** DZ4452C, 38.8 mm SL (5). **i** DZ4452A, 36.9 mm SL (5). **j** DZ5352B, 39.0 mm SL (2). **k** DZ5350E, 42.6 mm SL (4). **l** DZ5351I, 37.1 mm SL (3). **m** DZ5350G, 35.7 mm SL (4). **n** DZ5351A, 34.8 mm SL (3). **o** Live coloration of *P. nigrofasciata*. *Pethia nigrofasciata* in **p**–**r**. **p** DZ4403A, 39.1 mm SL, Kelani basin. **q** DZ4059B, 32.3 mm SL, Bentara basin. **r** DZ4509B, 44.2 mm SL, Walawe basin

**Fig. 2:**
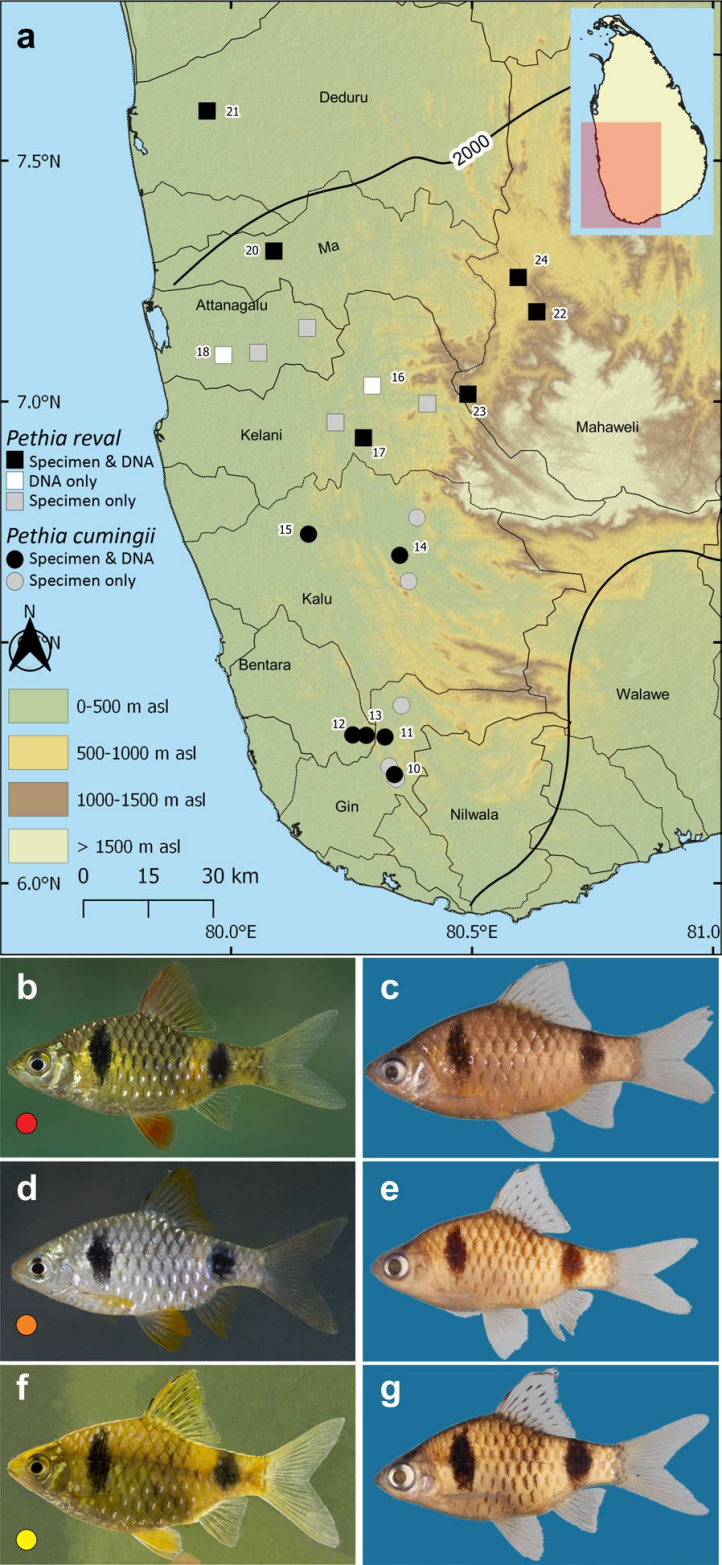
**a** Sri Lanka, showing the geographical origin of samples of *Pethia cumingii* and *P. reval* used in this study. The thin black lines indicate basin boundaries, while the bold black line indicates the 2000-mm isohyet, which encompasses the wet zone. Numbers on the map represent the sampling localities listed in Table 1. *Pethia reval* in **b** live coloration and **c** in preservation, DZ5354A, 30.9 mm SL, Attanagalu basin. Population of *P.* cf. *cumingii* from Kalu basin with orange fins in (**d**) live coloration and **e** in preservation, DZ3917A, 30.8 mm SL. *Pethia cumingii* from southerly basins (here Gin) in (**f**) live coloration and **g** in preservation, DZ5014A, 27.8 mm SL

**Fig. 3:**
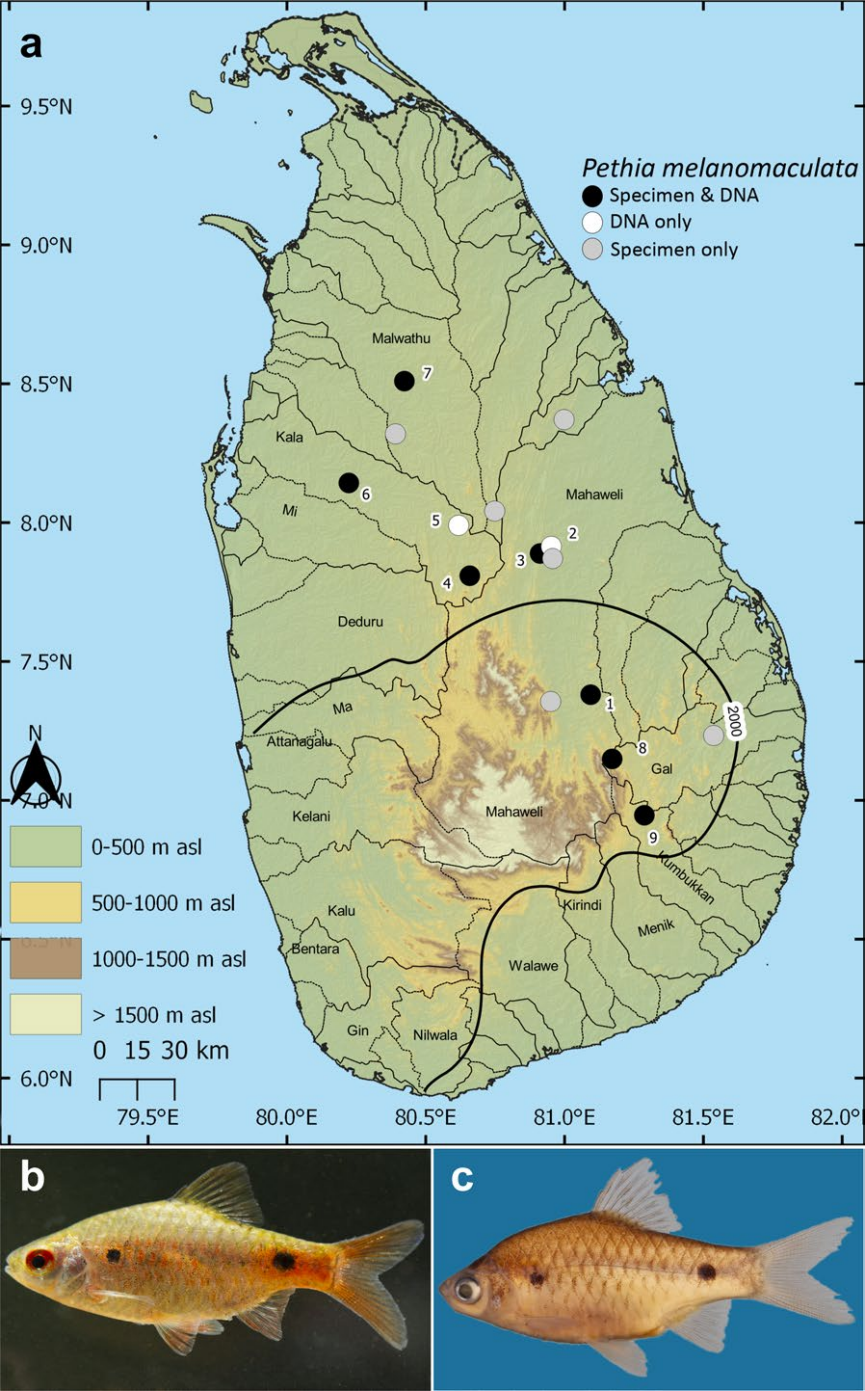
**a** Sri Lanka, showing the geographical origin of samples of *Pethia melanomaculata* used in this study. The thin black lines indicate basin boundaries, while the bold black line indicates the 2000-mm isohyet, which encompasses the wet zone. Numbers on the map represent the sampling localities listed in Table 1. *Pethia melanomaculata* in **b** live coloration and **c** in preservation, DZ4104, 34.1 mm SL, Mahaweli basin

Most studies that have referenced *Pethia* in South Asia up to now have involved new-species descriptions. Here, based on a sampling of 35 locations in 14 major river basins in Sri Lanka (Figs. 1a, 2a, 3a), and based on a mitochondrial and nuclear DNA dataset, we analyze, for the first time, the phylogeography and phylogenetic relationships of *Pethia* in the island in the context of representatives of the genus in India. Based on these, we evaluate the hypotheses set out above.

**Table 1:** Details of samples of Sri Lankan *Pethia* from which sequences were generated, with their localities, voucher references and GenBank accession numbers. LK, Sri Lanka

Voucher	Location	GPS coordinates	*cytb*	*cytb* haplotype	*rag1*	*rag1* haplotype
*Pethia melanomaculata*						
DZ1500	LK: Ulhitiya, Mahaweli (01)	7.3799 N 81.0939 E	MT732729	M3	MT732764	M1
DZ1501	LK: Ulhitiya, Mahaweli (01)	7.3799 N 81.0939 E	MZ686566	M3	–	–
DZ4302	LK: Angammedilla, Mahaweli (02)	7.9140 N 80.9520 E	MZ686567	M3	MZ686661	M1
DZ4303	LK: Angammedilla, Mahaweli (03)	7.8884 N 80.9116 E	MZ686568	M4	–	–
DZ4536	LK: Dambulu Oya, Dambulla, Kala (04)	7.8083 N 80.6584 E	MZ686569	M1	MZ686662	M1
DZ4793	LK: Maha Elagamuwa, Kala (05)	7.9898 N 80.6184 E	MZ686570	M1	MZ686663	M1
DZ4885	LK: Rajanganaya, Kala (06)	8.1415 N 80.2211 E	MZ686571	M1	MZ686664	M1
DZ5094	LK: Medawacchiya, Malwathu (07)	8.5091 N 80.4229 E	MZ686572	M1	MZ686665	M1
DZ5095	LK: Medawacchiya, Malwathu (07)	8.5091 N 80.4229 E	MZ686573	M2	–	–
DZ4570	LK: Kotagama, Gal (08)	7.1485 N 81.1715 E	MZ686574	M5	MZ686666	M2
DZ4583	LK: Bellan Oya, Kumbukkan (09)	6.9458 N 81.2874 E	MZ686575	M6	MZ686667	M1
DZ4584	LK: Bellan Oya, Kumbukkan (09)	6.9458 N 81.2874 E	MZ686576	M7	–	–
*Pethia cumingii*						
DZ5037	LK: Homadola, Gin (10)	6.2254 N 80.339 E	MZ686577	C9	MZ686668	R1
DZ5038	LK: Homadola, Gin (10)	6.2254 N 80.339 E	MZ686578	C9	–	–
DZ5039	LK: Homadola, Gin (10)	6.2254 N 80.339 E	MZ686579	C9	–	–
DZ4959	LK: Hiniduma, Gin (11)	6.3033 N 80.3193 E	MZ686580	C6	MZ686669	R1
DZ4960	LK: Hiniduma, Gin (11)	6.3033 N 80.3193 E	MZ686581	C8	MZ686670	R1
DZ4961	LK: Hiniduma, Gin (11)	6.3033 N 80.3193 E	MZ686582	C9	–	–
DZ4962	LK: Hiniduma, Gin (11)	6.3033 N 80.3193 E	MZ686583	C5	–	–
DZ4963	LK: Hiniduma, Gin (11)	6.3033 N 80.3193 E	MZ686584	C7	–	–
DZ3305	LK: Bambarawana, Mattaka, Bentara (12)	6.3076 N 80.2524 E	MT732731	C4	MT732766	R1
WHT01	LK: Bambarawana, Mattaka, Bentara (13)	6.3067 N 80.2804 E	MZ686585	C5	MZ686671	R1
DZ3026	LK: Ratnapura, Elapatha, Kalu (14)	6.6805 N 80.3499 E	MZ686586	C2	MZ686672	R1
DZ3056	LK: Ratnapura, Elapatha, Kalu (14)	6.6805 N 80.3499 E	MZ686587	C1	MZ686673	R4
DZ3117	LK: Dombagaskanda, Kalu (15)	6.7245 N 80.1606 E	MZ686588	C3	–	–
DZ3118	LK: Dombagaskanda, Kalu (15)	6.7245 N 80.1606 E	MZ686589	C2	MZ686674	R3
DZ3119	LK: Dombagaskanda, Kalu (15)	6.7245 N 80.1606 E	MZ686590	C1	MZ686675	R2
*Pethia reval*						
WHT03	LK: Yatiyanthota, Kelani (16)	7.0329 N 80.2929 E	MZ686591	R8	MZ686676	R1
DZ4999	LK: Yogama, Kelani (17)	6.9245 N 80.2746 E	MZ686592	R7	MZ686677	R1
DZ5000	LK: Yogama, Kelani (17)	6.9245 N 80.2746 E	MZ686593	R8	MZ686678	R1
DZ5001	LK: Yogama, Kelani (17)	6.9245 N 80.2746 E	MZ686594	R8	–	–
DZ5002	LK: Yogama, Kelani (17)	6.9245 N 80.2746 E	MZ686595	R8	–	–
DZ5003	LK: Yogama, Kelani (17)	6.9245 N 80.2746 E	MZ686596	R8	–	–
DZ3007	LK: Gampaha, Attanagalu (18)	7.0962 N 79.984 E	MZ686597	R5	–	–
DZ3091	LK: Gampaha, Attanagalu (18)	7.0962 N 79.984 E	MZ686598	R5	–	–
DZ5032	LK: Yakkala, Attanagalu (19)	7.0792 N 80.0732 E	MZ686599	R5	MZ686679	R1
DZ5033	LK: Yakkala, Attanagalu (19)	7.0792 N 80.0732 E	MZ686600	R5	MZ686680	R1
DZ5034	LK: Yakkala, Attanagalu (19)	7.0792 N 80.0732 E	MZ686601	R6	–	–
DZ5035	LK: Yakkala, Attanagalu (19)	7.0792 N 80.0732 E	MZ686602	C9	–	–
DZ5036	LK: Yakkala, Attanagalu (19)	7.0792 N 80.0732 E	MZ686603	C9	–	–
DZ4385	LK: Bopitiya, Giriulla, Ma (20)	7.3121 N 80.0888 E	MZ686604	R3	MZ686681	R1
DZ4386	LK: Bopitiya, Giriulla, Ma (20)	7.3121 N 80.0888 E	MZ686605	R4	MZ686682	R1
DZ4828	LK: Bopitiya, Giriulla, Ma (20)	7.3121 N 80.0888 E	MZ686606	R5	MZ686683	R1
DZ4829	LK: Bopitiya, Giriulla, Ma (20)	7.3121 N 80.0888 E	MZ686607	R3	MZ686684	R1
DZ4830	LK: Bopitiya, Giriulla, Ma (20)	7.3121 N 80.0888 E	MZ686608	R5	–	–
DZ3039	LK: Kolamunu Oya, Deduru (21)	7.6029 N 79.9502 E	MZ686609	R2	–	–
DZ3061	LK: Kolamunu Oya, Deduru (21)	7.6029 N 79.9502 E	MT732730	R1	MT732765	R1
DZ4823	LK: Nillamba, Hindagala, Mahaweli (22)	7.1861 N 80.6344 E	MZ686610	R11	–	–
DZ4824	LK: Nillamba, Hindagala, Mahaweli (22)	7.1861 N 80.6344 E	MZ686611	R11	–	–
DZ4825	LK: Nillamba, Hindagala, Mahaweli (22)	7.1861 N 80.6344 E	MZ686612	R11	–	–
DZ4826	LK: Nillamba, Hindagala, Mahaweli (22)	7.1861 N 80.6344 E	MZ686613	R11	–	–
DZ4827	LK: Nillamba, Hindagala, Mahaweli (22)	7.1861 N 80.6344 E	MZ686614	R10	–	–
DZ3238	LK: Ambagamuwa, Mahaweli (23)	7.0151 N 80.4915 E	MZ686615	R9	–	–
DZ3239	LK: Ambagamuwa, Mahaweli (23)	7.0151 N 80.4915 E	MZ686616	R8	MZ686685	R1
DZ3240	LK: Ambagamuwa, Mahaweli (23)	7.0151 N 80.4915 E	MZ686617	R8	MZ686686	R1
DZ3241	LK: Ambagamuwa, Mahaweli (23)	7.0151 N 80.4915 E	MZ686618	R8	–	–
DZ3256	LK: Ambagamuwa, Mahaweli (23)	7.0151 N 80.4915 E	MZ686619	R9	–	–
DZ3255	LK: Ambagamuwa, Mahaweli (23)	7.0151 N 80.4915 E	MZ686620	R9	–	–
DZ3257	LK: Ambagamuwa, Mahaweli (23)	7.0151 N 80.4915 E	MZ686621	R8	–	–
DZ3278	LK: Sarasavi Oya, Peradeniya, Mahaweli (24)	7.2574 N 80.5957 E	MZ686622	R9	MZ686687	R1
*Pethia nigrofasciata*						
DZ4449	LK: Alawala, Attanagalu (25)	7.12 N 80.1901 E	MZ686623	N1	MZ686688	N1
DZ4450	LK: Alawala, Attanagalu (25)	7.12 N 80.1901 E	MZ686624	N2	MZ686689	N1
DZ4451	LK: Alawala, Attanagalu (25)	7.12 N 80.1901 E	MZ686625	N3	–	–
DZ5040	LK: Alawala, Attanagalu (25)	7.12 N 80.1901 E	MZ686626	N1	MZ686690	N1
DZ5145	LK: Lenagala, Attanagalu (26)	7.1047 N 80.2177 E	MZ686627	N2	MZ686691	N1
DZ4343	LK: Illukowita, Thummodara, Kelani (27)	6.8796 N 80.1694 E	MZ686628	N4	MZ686692	N1
DZ4344	LK: Illukowita, Thummodara, Kelani (27)	6.8796 N 80.1694 E	MZ686629	N5	–	–
DZ3024	LK: Ratnapura, Elapatha, Kalu (14)	6.6805 N 80.3499 E	MZ686630	N6	–	–
DZ3025	LK: Ratnapura, Elapatha, Kalu (14)	6.6805 N 80.3499 E	MZ686631	N7	–	–
DZ3367	LK: Athwelthota, Kalu (28)	6.5382 N 80.2901 E	MZ686632	N9	MZ686693	N1
DZ4769	LK: Runakanda Forest, Kalu (29)	6.4548 N 80.3263 E	MZ686633	N8	MZ686694	N1
DZ4770	LK: Runakanda Forest, Kalu (29)	6.4548 N 80.3263 E	MZ686634	N8	–	–
DZ3095	LK: Thundola, Horawala, Bentara (30)	6.475 N 80.1524 E	MT732732	N11	MT732767	N4
DZ3096	LK: Thundola, Horawala, Bentara (30)	6.475 N 80.1524 E	MZ686635	N10	MZ686695	N4
DZ4964	LK: Hiniduma, Gin (11)	6.3033 N 80.3193 E	MZ686636	N12	MZ686696	N2
DZ4965	LK: Hiniduma, Gin (11)	6.3033 N 80.3193 E	MZ686637	N13	–	–
DZ4928	LK: Kottawa Forest, Galle, Gin (31)	6.0986 N 80.3146 E	MZ686638	N14	MZ686697	N3
DZ4929	LK: Kottawa Forest, Galle, Gin (31)	6.0986 N 80.3146 E	MZ686639	N14	–	–
DZ4930	LK: Kottawa Forest, Galle, Gin (31)	6.0986 N 80.3146 E	MZ686640	N15	–	–
DZ4478	LK: Ugudu dola, Opatha, Nilwala (32)	6.2669 N 80.4023 E	MZ686641	N17	MZ686698	N2
DZ4479	LK: Ugudu dola, Opatha, Nilwala (32)	6.2669 N 80.4023 E	MZ686642	N18	MZ686699	N2
DZ5060	LK: Kotapola, Nilwala (33)	6.2949 N 80.544 E	MZ686643	N16	MZ686700	N1
DZ5061	LK: Kotapola, Nilwala (33)	6.2949 N 80.544 E	MZ686644	N16	MZ686701	N1
DZ5062	LK: Kotapola, Nilwala (33)	6.2949 N 80.544 E	MZ686645	N16	–	–
DZ1454	LK: Pambahinna, Walawe (34)	6.7018 N 80.8006 E	MZ686646	N19	–	–
DZ4244	LK: Rajawaka, near Kaltota, Walawe (35)	6.6064 N 80.8195 E	MZ686647	N19	MZ686702	N2
DZ4245	LK: Rajawaka, near Kaltota, Walawe (35)	6.6064 N 80.8195 E	MZ686648	N19	MZ686703	N1
DZ3233	LK: Ambagamuwa, Mahaweli (23)	7.0151 N 80.4915 E	MZ686649	N6	MZ686704	N1
DZ3234	LK: Ambagamuwa, Mahaweli (23)	7.0151 N 80.4915 E	MZ686650	N23	–	–
DZ3236	LK: Ambagamuwa, Mahaweli (23)	7.0151 N 80.4915 E	MZ686651	N22	–	–
DZ3237	LK: Ambagamuwa, Mahaweli (23)	7.0151 N 80.4915 E	MZ686652	N5	–	–
DZ3263	LK: Ambagamuwa, Mahaweli (23)	7.0151 N 80.4915 E	MZ686653	N5	–	–
DZ3264	LK: Ambagamuwa, Mahaweli (23)	7.0151 N 80.4915 E	MZ686654	N23	–	–
DZ3265	LK: Ambagamuwa, Mahaweli (23)	7.0151 N 80.4915 E	MZ686655	N20	–	–
DZ3266	LK: Ambagamuwa, Mahaweli (23)	7.0151 N 80.4915 E	MZ686656	N6	–	–
DZ3267	LK: Ambagamuwa, Mahaweli (23)	7.0151 N 80.4915 E	MZ686657	N21	–	–
DZ3268	LK: Ambagamuwa, Mahaweli (23)	7.0151 N 80.4915 E	MZ686658	N23	–	–
DZ3269	LK: Ambagamuwa, Mahaweli (23)	7.0151 N 80.4915 E	MZ686659	N20	–	–
DZ3270	LK: Ambagamuwa, Mahaweli (23)	7.0151 N 80.4915 E	MZ686660	N20	–	–

Further, several translocated populations of *P. nigrofasciata* and *P. reval* are documented from the Mahaweli basin in the Central Hills [22, 32–34]. However, the origin of the translocated stock has until now not been established. Here, using our molecular dataset representative of both translocated and native populations of these two species, we seek to establish the provenance of these introduced populations. Finally, based on both molecular and morphometric data, we investigate the species-diversity of *Pethia* in Sri Lanka following the general-lineage concept of species [35], and delineate the geographic distributions of the species.

## Results

### Molecular phylogeny

**Fig. 4:**
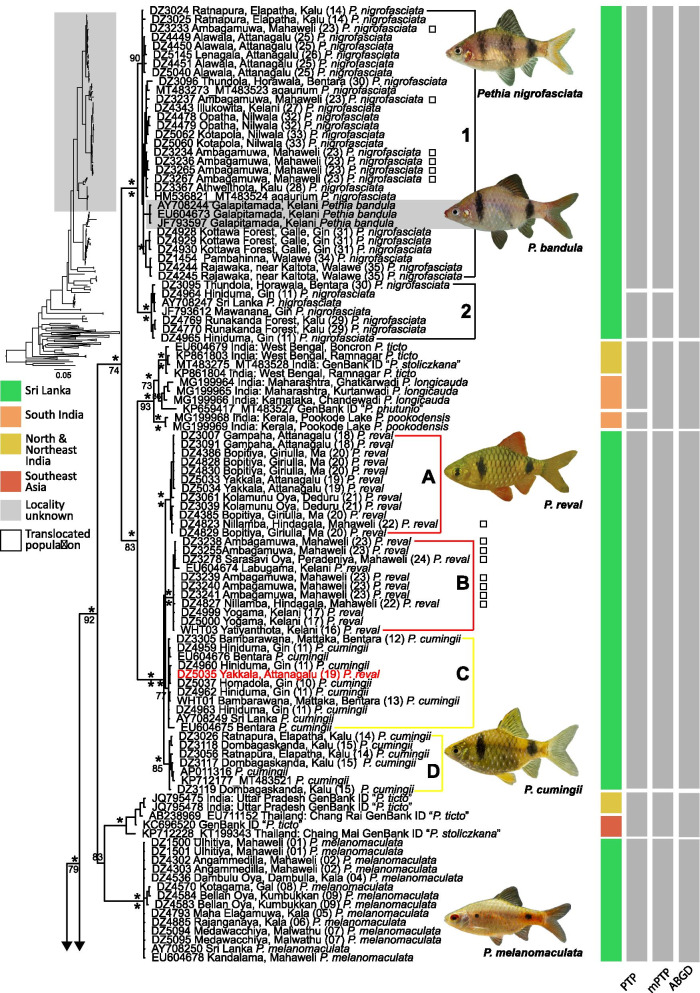

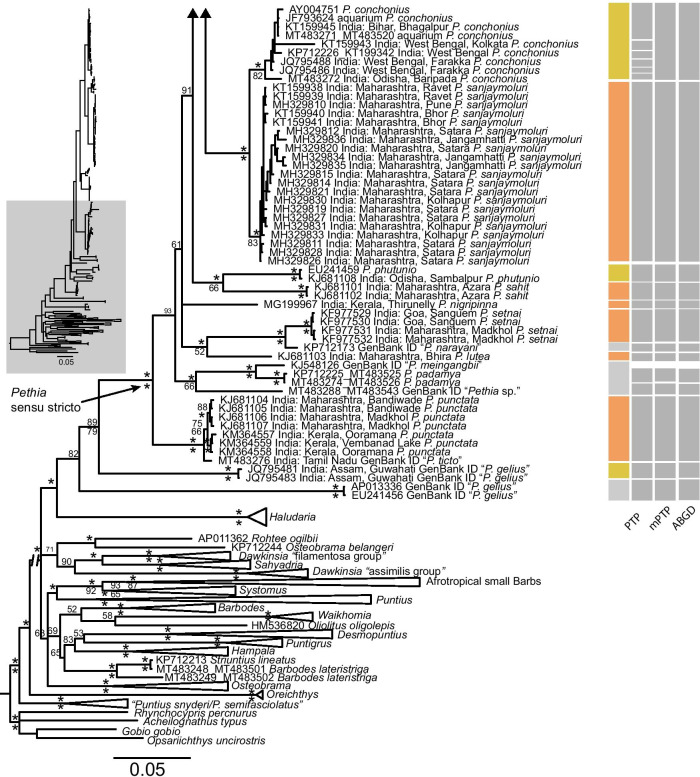
Molecular phylogenetic relationships of *Pethia*, based on Bayesian inference of the concatenated *cytb* + *rag1* (2572 bp) data set. Asterisks (*) above and below nodes represent ≥ 95% Bayesian posterior probabilities and ML bootstrap values, respectively. Scale bar represents number of changes per site. Node support below 50 is not labeled. Results of the molecular species delimitation methods (PTP, mPTP and ABGD) for *cytb* are shown as grey rectangles on the right. The results of the ABGD shown are based on the initial partition using the p-distance model at p =  ~ 0.01 and X = 1.5. The GenBank sample “*P. meingangbii*” is represented by only a *rag1* sequence (KJ548126) and is not included in the species delimitation analyses. In the mPTP analysis, *P. nigripinna* (MG199967) and “*Pethia* sp.” (MT483288) were delimited as a single species

The *cytb* and *rag1* phylogenetic analyses recovered mostly similar topologies with respect to the relationships of Sri Lankan species of *Pethia* in both the ML and the BI frameworks, with differences observed mainly in branch lengths. Here we focus on the concatenated *cytb* + *rag1* dataset (Fig. 4). Differences between the single-gene and concatenated datasets are mentioned, where necessary.

While the monophyly of *Pethia* is supported, with strong node support, in all the analyses (Fig. 4), the sister-group relationships of the genus are not clearly resolved. The South Indian genus *Haludaria* is recovered as the sister group of *Pethia* in the concatenated *cytb* + *rag1* BI and ML phylogenies (PP = 98, BP < 50), and the *cytb* BI phylogeny (PP = 70), with moderate to weak node support (Fig. 4, Additional file 1: Fig. S1). In the *cytb* ML phylogeny, the clade of *Systomus* + Afrotropical small barbs is recovered as the sister group of *Pethia,* but with weak node support (BP < 50). In the *rag1* BI and ML phylogenies, the sister group of *Pethia* is a clade of Southeast Asian barbs represented by the genera *Barbodes*, *Desmopuntius*, *Hampala*, *Puntigrus*, *Oliotius*, and *Striuntius,* but with weak node support (PP = 78, BP < 50, Additional file 1: Fig. S2).

The Sri Lankan species of *Pethia* are recovered as polyphyletic in all the analyses (Fig. 4, Additional file 1: Figs. S1, S2). A clade represented by GenBank sequences of *Pethia* from Uttar Pradesh (North India) and Thailand (Southeast Asia), identified either as “*P. ticto”* or “*P. stoliczkana”,* is recovered as the sister group of *Pethia melanomaculata* in the BI and ML analysis of the concatenated (PP = 83, BP < 50) and BI *cytb* phylogenies (PP < 50), but with weak node support (Fig. 4, Additional file 1: S1). In the ML *cytb* phylogeny, *P. melanomaculata* is recovered as the sister group of the clades that include *P. nigrofasciata*, *P. cumingii*, *P. reval*, *P. ticto*, *P. longicauda* and *P. pookodensis,* but with weak node support (BP < 50). In the *rag1* phylogeny, the GenBank sequence identified as “*P. stoliczkana”* is recovered as the sister group of *Pethia melanomaculata,* with strong node support (Additional file 1: Fig. S2).

A clade comprising *P. longicauda* and *P. pookodensis* from peninsular India, and *P. ticto *sensu stricto from North India, is recovered as the sister group of the clade that includes the Sri Lankan *P. reval* and *P. cumingii* in the concatenated and *cytb* phylogenies, with strong node support (Fig. 4, Additional file 1: S1). In the *rag1* phylogeny, a clade comprising of GenBank sequences identified as “*P. phutunio”* (= *P. longicauda*), “*P. stoliczkana”* (= *P. ticto*), or “*P. ticto”* is recovered as the sister group of the clade that includes the Sri Lankan *P. reval* and *P. cumingii,* with strong node support (Additional file 1: Fig. S2). *Pethia cumingii* and *P. reval* are not recovered as reciprocally monophyletic. Four subclades which, for brevity, we name A, B, C, and D, are recognized within *P. cumingii* and *P. reval* in the concatenated and *cytb* phylogenies, with strong node support (Fig. 4, Additional file 1: Figs. S1, S2). Among these four subclades, A and B are representative of *P. reval*, while C and D are mostly representative of *P. cumingii,* based on the geographic distribution and coloration of the specimens sequenced. Subclade A consists of *P. reval* originating from the Attanagalu, Ma and Deduru basins, while subclade B consists of *P. reval* originating from the Kelani basin. The translocated populations of *P. reval* in the Mahaweli are represented in both subclades A and B. Subclade C includes *P. cumingii* originating from the Bentara and Gin basins and *P. reval* from the Attanagalu basin (Fig. 4, Additional file 1: Fig. S1). Subclade D includes *P. cumingii* originating from the Kalu basin. Such geographic structure in populations of *P. reval* and *P. cumingii* is not evident from the *rag1* phylogeny (Additional file 1: Fig. S2).

In the *cytb* and the concatenated phylogeny, the clade that includes *Pethia nigrofasciata* and *P. bandula* is recovered, with strong node support, as the sister group of the clade that includes *P. cumingii*, *P. reval*, *P. ticto*, *P. longicauda* and *P. pookodensis* (Fig. 4, Additional file 1: Fig. S1). Within *P. nigrofasciata*, two subclades are recovered in the concatenated and the *cytb* phylogenies (subclades 1 and 2; Fig. 4, Additional file 1: Fig. S1). Subclade 1 is a widespread lineage represented by samples from the Attanagalu, Kelani, Kalu, Bentara, Gin, Nilwala and Walawe basins. Subclade 2 is a lineage represented by samples from the Kalu, Bentara and Gin basins. The samples DZ3096 and DZ3095 sequenced from Horawala in the Bentara basin belong to subclades 1 and 2, respectively. The remaining locations from which *P. nigrofasciata* have been sequenced are not shared between these two subclades (Fig. 4, Additional file 1: Fig. S1). All the populations of *P. nigrofasciata* translocated to the Mahaweli basin belong to subclade 1. In the *rag1* phylogeny, these two subclades are not apparent in *P. nigrofasciata* (Additional file 1: Fig. S2). The phylogenetic relationships of *P. bandula* are not clearly resolved: it is clustered within subclade 1 of *P. nigrofasciata* (Fig. 4, Additional file 1: Fig. S1).

### Molecular species delimitation

The number of species delimited in PTP, mPTP and ABGD were mostly congruent (Fig. 4). The MCMC analyses for both PTP and mPTP reached convergence based on the plots of generation vs. likelihood score. For the 145-taxon dataset for *cytb*, the mPTP and the PTP analyses delimited 18 and 25 species, respectively (Fig. 4). In both analyses, the posterior support from the MCMC analyses for most species delimited was 1.0. The main difference between the outcomes of these two analyses was that in the PTP analysis, the clade that included *P. conchonius* was unrealistically delimited as six species, whereas the mPTP analysis delimited this clade as a single species (Fig. 4). In the ABGD analysis, at a minimum gap width of 1.5, 1.0 or 0.8 and a p-value of ~ 0.01, the initial partition of the p-distance, and JC69 models, inferred 17 species while the K80 model inferred 14 species.

The subclades 1 and 2 of *P. nigrofasciata* were delimited as two species in the PTP and mPTP analysis, while ABGD delimited these as a single species. *Pethia bandula,* which is not resolved in the molecular phylogeny, was delimited as conspecific with *P. nigrofasciata* (Fig. 4). *Pethia cumingii* and *P. reval* were delimited as a single species in all three analyses, while *Pethia melanomaculata* too, was delimited as a single species in all three analyses. Among the Indian taxa, most valid species were correctly delimited. The most notable exception was the clade that includes *P. ticto*, *P. longicauda* and *P. pookodensis*. All three molecular species-delimitation methods failed to delimit *ticto* and *P. longicauda,* while *P. pookodensis* was delimited only in the PTP analysis (Fig. 4).

The uncorrected pairwise *cytb* genetic distances for Sri Lankan species of *Pethia* are given in Additional file 1: Table S5. The smallest maximum intraspecific genetic distance in *cytb* among these species is 0.6%, in *P. melanomaculata,* while the greatest is 4.0%, between subclades 1 and 2 in *P. nigrofasciata*. *Pethia melanomaculata* differs from the members of the clade that is recovered as its sister group in the concatenated phylogeny by an uncorrected pairwise *cytb* distance of 4.4–6.4%, while *P. cumingii* and *P. reval* differ from their sister group, which includes *P. ticto*, *P. longicauda* and *P. pookodensis,* by 4.7–6.9%.

### Genetic diversity and phylogeography

For each gene marker (*cytb* and *rag1*), the number of haplotypes, polymorphic sites, parsimony-informative sites, and nucleotide and haplotype diversities, are given in Additional file 1: Table S6 for *P. cumingii*, *P. melanomaculata*, *P. nigrofasciata*, and *P. reval*. Overall, the nucleotide and haplotype diversities among the wet zone species (*P. nigrofasciata*, *P. cumingii* and *P. reval*) were greater compared to those of the dry zone species, *P. melanomaculata*. None of the neutrality tests were significant for any of these four species (Additional file 1: Table S6).

**Fig. 5:**
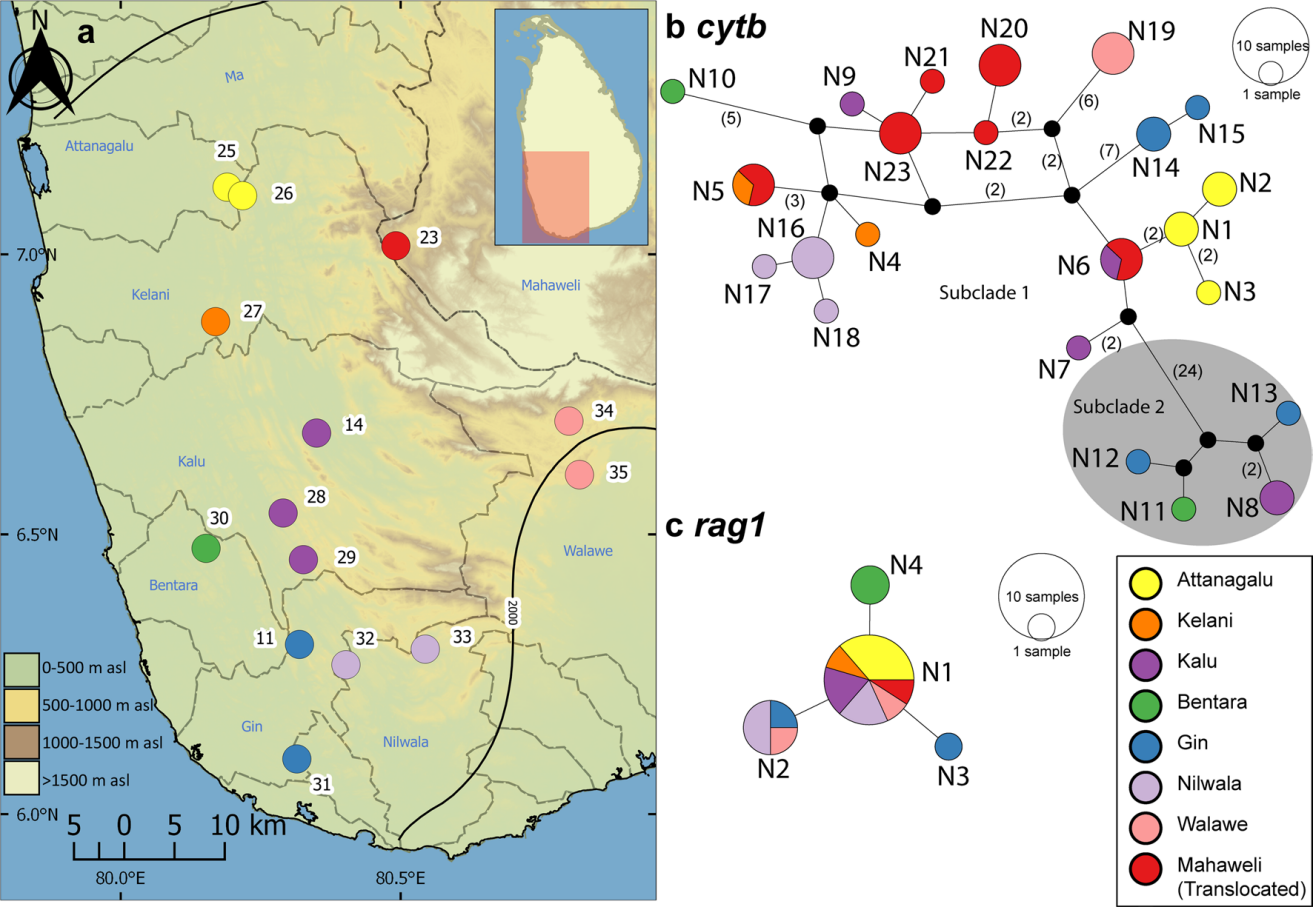
**a** Sampling localities for *Pethia nigrofasciata* for the molecular analysis in the present study. Numbers on the map represent the sampling localities listed in Table 1. Median-joining haplotype network for *P. nigrofasciata*, based on the analysis of **b** a 1082 bp fragment of the *cytb* gene, and **c** a 1490 bp fragment of the *rag1* gene. The number of mutational steps > 1 is indicated in parentheses. The black circles are hypothetical nodes. Legend colours correspond to river basins

In the *cytb* haplotype network for *P. nigrofasciata*, only two haplotypes (N5 and N6) are shared between basins, while the remainder are confined to individual basins (Fig. 5b). The haplotypes N5 and N6 occur in translocated populations of *P. nigrofasciata* in the Mahaweli basin; these haplotypes are shared with the Kelani and Kalu basins, respectively. Four more haplotypes N20-N23 from the translocated populations in the Mahaweli basin form unique haplotypes. While most haplotypes are confined to individual basins, they do not show a clear phylogeographic structure in the median-joining haplotype network. Subclade 2 of *P. nigrofasciata* is separated from subclade 1 by a minimum of 27 mutation steps. The *rag1* median-joining haplotype network for *P. nigrofasciata* exhibits a star-like pattern in which N1, a high-frequency haplotype, is surrounded by low-frequency haplotypes, each separated from N1 by a single mutational step (Fig. 5c).

**Fig. 6:**
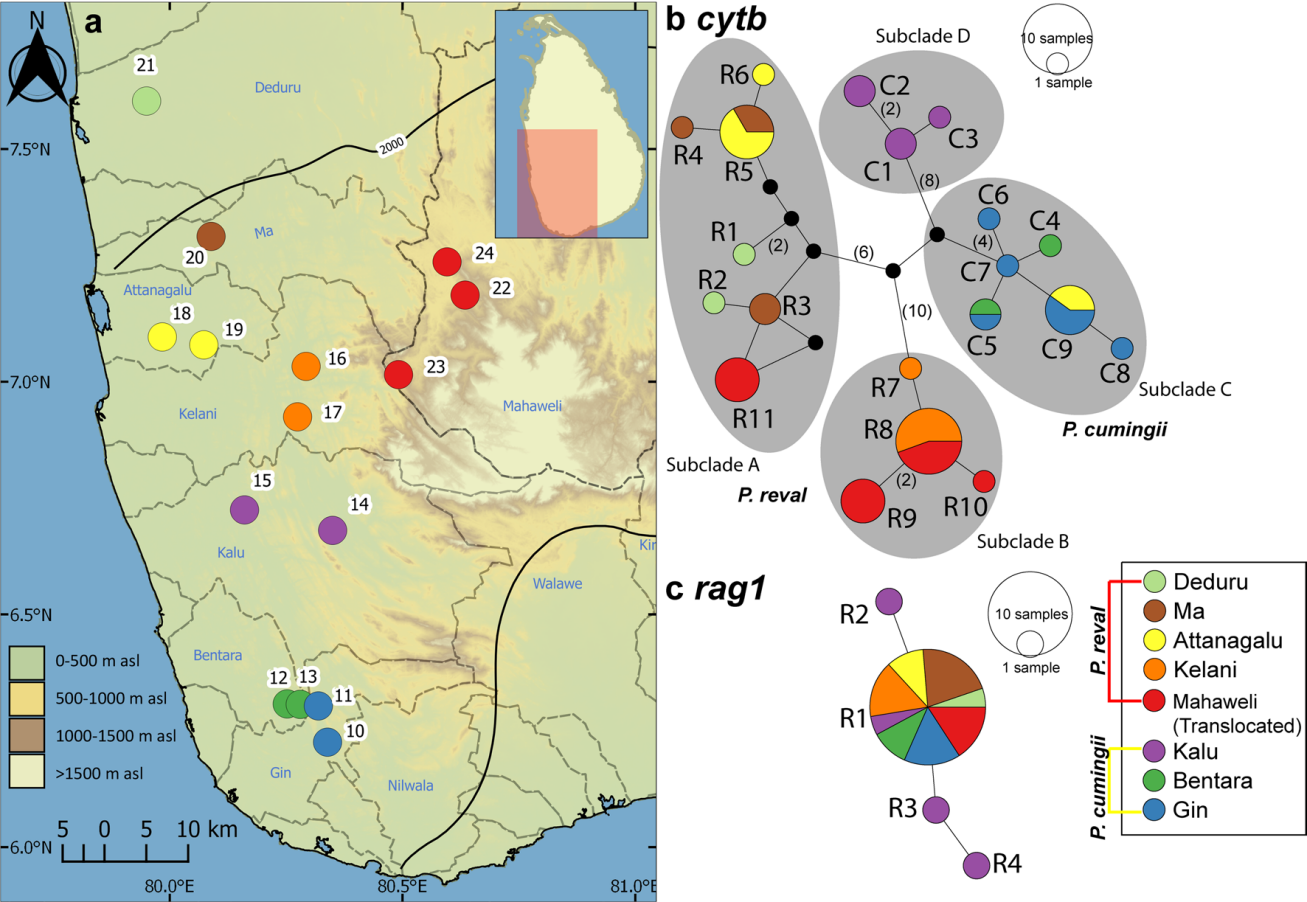
**a** Sampling localities for *Pethia cumingii* and *P. reval* for the molecular analysis in the present study. Numbers on the map represent the sampling localities listed in Table 1. Median-joining haplotype network for *Pethia cumingii* and *P. reval*, based on the analysis of **b** a 1082 bp fragment of the *cytb* gene, and **c** a 1490 bp fragment of the *rag1* gene. The number of mutational steps > 1 is indicated in parentheses. The black circles are hypothetical nodes. Legend colours correspond to river basins

In the *cytb* haplotype network for *P. cumingii* and *P. reval*, the four subclades are separated from each other by a minimum of 12 mutational steps (Fig. 6b). Only four haplotypes (R5, R8, C5 and C9) are shared between basins; the rest are confined to the individual basins (Fig. 6b). Haplotype R5 is shared between a pair of adjacent basins, the Ma and Attanagalu, while haplotype R8 is shared between the Kelani and the translocated populations of *P. reval* in the Mahaweli basin. Haplotype C5 is shared between the two adjacent basins, Bentara and Gin, while C9 is the only shared haplotype between *P. cumingii* (from the Gin basin) and *P. reval* (from the Attanagalu basin). Three other haplotypes, R9-R10 and R11 identified in the translocated populations in the Mahaweli basin, form unique haplotypes within subclades B and A, respectively (Fig. 6b). There is some phylogeographic structure in *P. cumingii* and *P. reval,* which is also reflected in the four subclades. Excluding the populations translocated to the Mahaweli, in subclade A, *P. reval* is represented in samples from the Deduru, Ma, and Attanagalu basins, while in subclade B, it is represented in samples from the Kelani basin. Subclade D contains only samples from the Kalu basin, while in subclade C, except for C9, the remaining haplotypes are confined to the southern Bentara and Gin basins (Fig. 6b). The *rag1* median-joining haplotype network for *P. cumingii* and *P. reval* exhibit a high-frequency haplotype, R1, found in both *P. cumingii* and *P. reval* from all the sampled river basins, in addition to three haplotypes, R2-R4, unique to the Kalu basin (Fig. 6c).

**Fig. 7:**
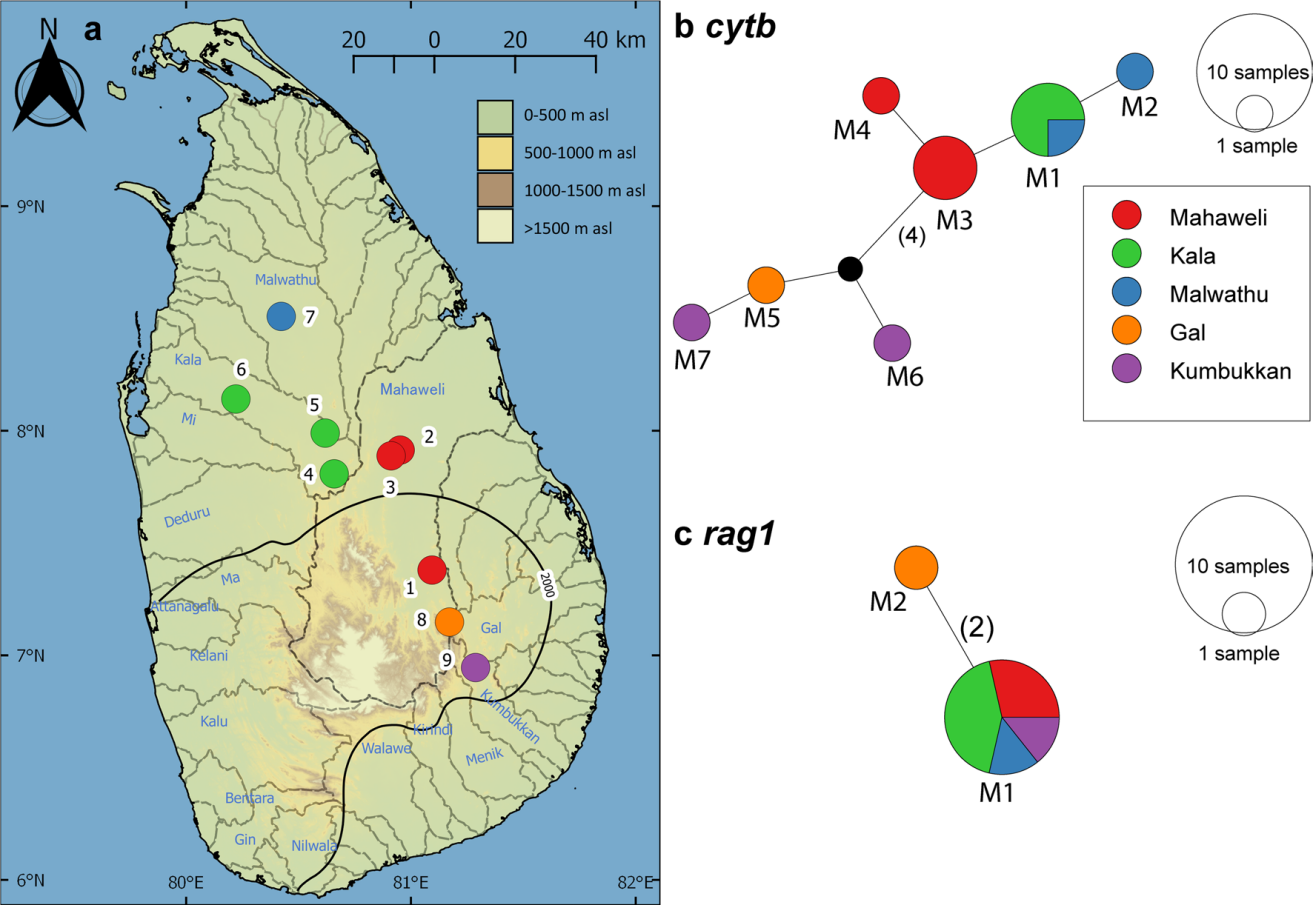
**a** Sampling localities for *Pethia melanomaculata* for the molecular analysis in the present study. Numbers on the map represent the sampling localities listed in Table 1. Median-joining haplotype network for *P. melanomaculata*, based on the analysis of (**b**) a 1082 bp fragment of the *cytb* gene, and **c** a 1490 bp fragment of the *rag1* gene. The number of mutational steps > 1 is indicated in parentheses. The black circles are hypothetical nodes. Legend colours correspond to river basins

In the *cytb* haplotype network for *P. melanomaculata*, only a single haplotype, M1, is shared between two adjacent basins: Kala and Malwathu, in the northern dry zone. The rest are confined to individual basins (Fig. 7b). The haplotypes from the eastern basins Gal (M5) and Kumbukkan (M6 and M7) are separated from those of the Mahaweli and northern dry zone basins by a minimum of five mutational steps (Fig. 7b). The *rag1* haplotype network for *P. melanomaculata* contains only two haplotypes, M1 and M2, in which M1 is a high-frequency haplotype, while M2 is confined to the Gal basin (Fig. 7c).

### Morphometry, meristics and statistical analysis

**Fig. 8:**
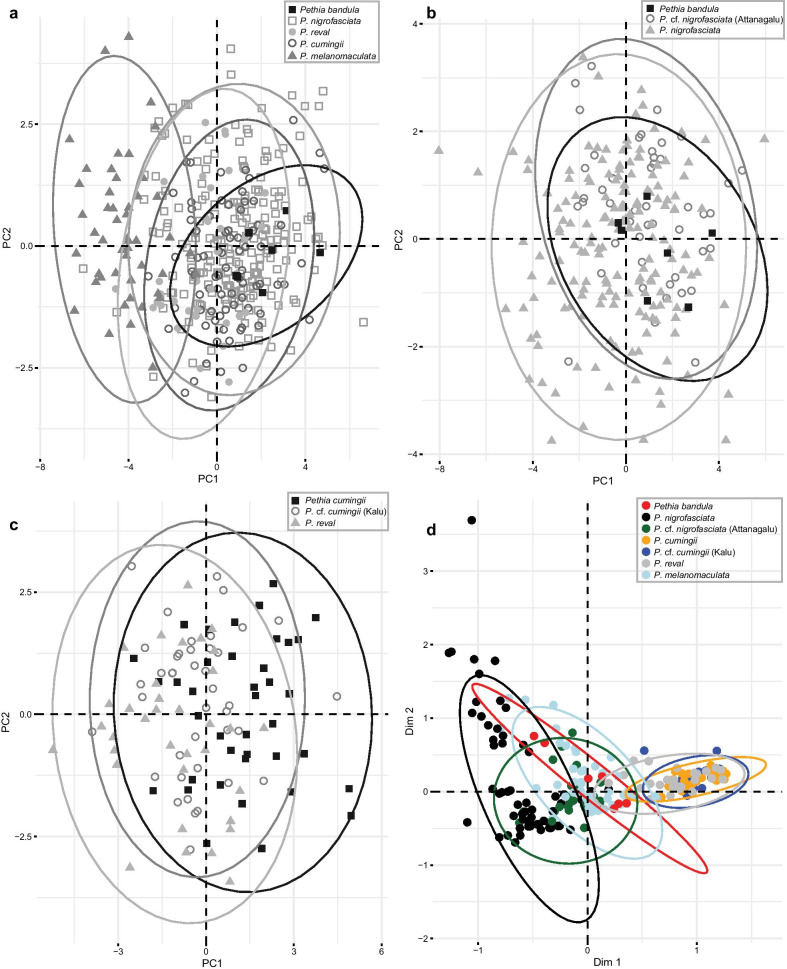
Multivariate analysis of morphometric data for the Sri Lankan species of *Pethia*. **a** PCA biplot of factor scores and factor loadings for *P. bandula*, *P. nigrofasciata*, *P. cumingii*, *P. reval*, and *P. melanomaculata*. **b** PCA biplot of factor scores and factor loadings for *P. bandula*, *P. nigrofasciata* (excluding Attanagalu population), and *P.* cf. *nigrofasciata* (Attanagalu population). **c** PCA biplot of factor scores and factor loadings for *P. reval*, *P. cumingii* (excluding Kalu population), and *P.* cf. *cumingii* (Kalu population). **d** MCA biplot of factor scores and factor loadings for meristic data in *P. bandula*, *P. nigrofasciata* (excluding Attanagalu population), *P.* cf. *nigrofasciata* (Attanagalu population), *P. cumingii* (excluding Kalu population), *P.* cf. *cumingii* (Kalu population), *P. reval*, and *P. melanomaculata*. Ellipses delineate 95% confidence intervals

**Fig. 9:**
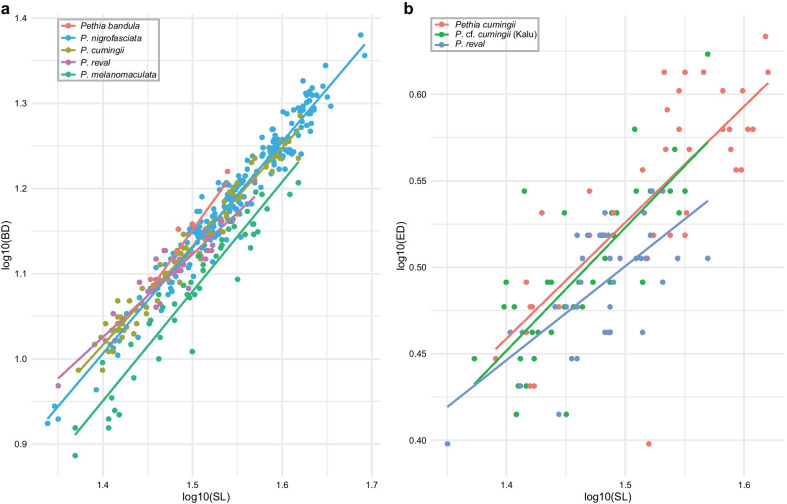
Relationship of **a** body depth in the Sri Lankan species of *Pethia* and **b** eye diameter in *P. reval*, *P. cumingii* (excluding the Kalu-basin population), and *P.* cf. *cumingii* (Kalu-basin population) vs standard length

In the size corrected PCA of morphometric measurements of the five species of *Pethia* represented in Sri Lanka, PC1 and PC2 explain 41.5% of the total variance (Fig. 8a). In this PCA, PC1 is explained mostly by body depth and interorbital width, while PC2 is explained mostly by postdorsal length and prepelvic length (Additional file 1: Table S7). *Pethia bandula*, *P. nigrofasciata*, *P. cumingii*, and *P. reval* show almost complete overlap in the morphospace, while *P. melanomaculata* shows a separation from the preceding four species (Fig. 8a). The relationship between body depth and standard length in these five species is illustrated in Fig. 9a. *Pethia melanomaculata* has a lesser body depth (32.3–42.2% SL, mean = 38.0) compared with *P. bandula* (42.2–48.0% SL, mean = 44.5), *P. nigrofasciata* (37.2–50.5% SL, mean = 44.0), *P. cumingii* (38.6–46.4% SL, mean = 42.8), and *P. reval* (38.7–45.6% SL, mean = 42.0). Similarly, *P. melanomaculata* also has a lesser head depth (19.5–23.9% SL, mean = 21.9) compared with *P. bandula* (23.1–26.1% SL, mean = 24.6), *P. nigrofasciata* (21.1–26.6% SL, mean = 23.6), *P. cumingii* (21.4–26.9% SL, mean = 23.7), and *P. reval* (21.1–25.2% SL, mean = 23.4). The other measurements mostly overlap among the five species (Additional file 1: Table S8).

The populations of *P. nigrofasciata* from the headwaters of the Attanagalu basin proximal to the type locality of *P. bandula* show a mix of meristic characters and intermediate color patterns between *P. nigrofasciata* and *P. bandula* (Fig. 1g–n, Additional file 1: Table S9). The specimens in the type series of *P. bandula* (n = 7) possess an incomplete lateral line with 7 (2), 8 (1), 9 (3), or 10 (1) pored scales in the lateral-line series (Fig. 1d–f). Except for a single specimen, the series of *P. nigrofasciata* (n = 167) examined from all the river basins excluding Attanagalu possess a complete lateral line, with 17 (1), 18 (9), 19 (62), 20 (64), 21 (23), or 22 (7) pored scales (Fig. 1p–r). In contrast, 30 specimens from populations of *P. nigrofasciata* in the headwaters of the Attanagalu basin possessed an incomplete lateral line, with 6 (5), 7 (2), 8 (6), 9 (3), 10 (2), 11 (4), 12 (3), 13 (2), 15 (1), or 16 (2) pored lateral-line scales (Fig. 1i–l), while a further 17 possess a complete lateral line with 19 (6), 20 (9), or 21 (2) pored scales (Fig. 1g, h, m, n, Additional file 1: Table S9). The color pattern of *P. bandula* includes two bars: one on the humeral-cleithral region, and another at the base of the caudal peduncle, whereas in *P. nigrofasciata*, in addition to the above bars, there is a further, wider bar beneath the dorsal-fin base. However, among the populations of *P. nigrofasciata* from the Attanagalu basin, in 19 specimens, the wide bar beneath the dorsal fin was incomplete (Fig. 1h–j), or absent (Fig. 1g), while in the remaining 28 specimens it was complete (Fig. 1k–n). In the size corrected PCA of the morphometric measurements of *P. bandula*, *P. nigrofasciata* (excluding Attanagalu populations), and *P.* cf. *nigrofasciata* (Attanagalu population) did not show a clear separation in morphospace (Fig. 8b): the three groups overlapped one another (Additional file 1: Table S10).

*Pethia reval* from the Kelani to the Deduru basins possess red dorsal, pelvic, and anal fins (Fig. 2b), while in *P. cumingii* from the Bentara and Gin, these fins are yellow (Fig. 2f). However, in populations of *P. cumingii* from the Kalu basin, the dorsal, pelvic, and anal fins are orange (Fig. 2d). Ref. [28] hypothesized that the Kalu basin may have been a zone of hybridization between red-finned *P. reval* and yellow-finned *P. cumingii.* To test whether these three groups (i.e., *P. reval*, the Bentara and Gin populations of *P. cumingii*, and the Kalu population of *P.* cf. *cumingii*), separate in the morphospace, we carried out a size-corrected PCA of morphometric measurements, which showed almost a complete overlap among the three groups (Fig. 8c). The relationship between eye diameter and standard length, which was identified by Meegaskumbura et al. [28] to be of value in distinguishing between *P. reval* (present study: eye diameter 8.6–11.5% SL, mean = 10.3) and *P. cumingii* (present study: 7.6–12.6% SL, mean = 10.5), remains generally consistent when a large series of specimens is considered, although with a greater variation (Fig. 9b). In *P.* cf. *cumingii,* the relationship between eye diameter and standard length (present study: 9.2–13.5% SL, mean = 10.8) is similar to that of *P. cumingii*. The remaining measurements show a broad overlap among these three groups (Additional file 1: Table S11).

In the MCA for the meristic data, the first and second dimensions explain 13.9% of the total variance (Fig. 8d). In this MCA, the first dimension is explained mostly by the condition of the lateral line (complete or incomplete), the number of scales between the lateral line and dorsal-fin origin, and the number of circumpeduncular scales, while the second dimension is explained mostly by the number of pored scales in the lateral line and the number of scales along the lateral series. In the MCA, *P. reval*, *P. cumingii*, and *P.* cf. *cumingii* form a cluster with almost complete overlap. *Pethia bandula*, *P. nigrofasciata*, and *P.* cf. *nigrofasciata* form another cluster with partial overlap with each other, though separated from *P. reval*, *P. cumingii*, and *P.* cf. *cumingii* (Fig. 8d). *Pethia melanomaculata* show overlaps partially with both the aforementioned clusters.

## Discussion

### The genus *Pethia*

Following the recognition of the genus by Pethiyagoda et al. [16], several studies confirmed the monophyly of *Pethia* [e.g., 10, 11, 18, 25, 26]. Nevertheless, about half of the 43 species included in the genus since 2012 are yet to be represented in a molecular phylogenetic framework. Doubt remains as to the generic placement of several of these. While Pethiyagoda et al. [16] allocated 23 species to the genus, they drew attention to others for which insufficient information was available, upon which to make a generic placement. ‘*Pethia’ narayani, ‘P.’ sharmai, ‘P.’ aurea, ‘P.’ cania*, ‘*P.’ gelius*, ‘*P*.’ *guganio, ‘P. castor’,* and *‘P. pollux’* are morphologically so distinct from *Pethia* sensu stricto and other Smiliogastrini that they may warrant placement in different genera [16, 18, 19]. The original description by Hora [36] of ‘*Pethia’ narayani* does not mention a serrated last unbranched dorsal-fin ray, which is a synapomorphy in *Pethia.* Hora [36] mentions only that “The dorsal fin… possesses a feeble and articulated spine which is considerably longer than the head; its free border is slightly concave though rounded at the top.” The holotype of *‘P.’ narayani*, illustrated in Hora [36], exhibits three vertical bands on the side of the body. ‘*Pethia’ narayani* in fact may belong to the recently described smiliogastrine genus *Waikhomia* [37]. ‘*Pethia’ sharmai* differs from all congeners in having 40 or more scales along the lateral-line row (vs. 30 or less in *Pethia* sensu stricto: see Additional file 1: Table S1). It superficially resembles ‘*P*.’ *guganio*. *‘Pethia’ aurea, ‘P.’ cania* and *‘P.’ gelius* appear to form a closely related group united by a striking colour pattern, distinguishing the group from *Pethia* s.s. [38]. In fact, even in our phylogenies, the GenBank sequences identified as “*P. gelius*” formed a distinct lineage basal to *Pethia* s.s. (Fig. 4). It seems possible also that ‘*P*.’ *castor*, and ‘*P*.’ *pollux* [18], from Myanmar, may belong to a lineage distinct from *Pethia*. The phylogenetic relationships of the mentioned Indian and Myanmarese species remain to be explored.

The type species of *Pethia, P. nigrofasciata*, a Sri Lankan endemic, is included in the present study. Consistent with this, the 36 species we treat as belonging to *Pethia* s.s. (Additional file 1: Table S1) are characterized by having the following suite of characters: 8 branched dorsal-fin rays; 5 branched anal-fin rays; the last unbranched dorsal-fin ray strong, serrated on its posterior margin; 2 or 3 black spots, blotches or bands laterally, including one on the humeral-cleithral region and another above the anal fin or on the caudal peduncle; the lateral line more often incomplete (26 species) than complete (10 species); barbels usually absent (32 species); and 19–30 scales in the lateral-line series. The maximum size for the genus is usually < 50 mm SL [16]. Most species (perhaps all) are sexually dichromatic. All five species of *Pethia* in Sri Lanka are consistent with the above conception of the genus.

### Phylogeny

Our taxon sampling includes all five Sri Lankan species of *Pethia*, sampled from the island’s principal rivers, including *cytb* sequences derived from reliably identified specimens for *P. bandula* as well as almost all the species reported from South India, to which the Sri Lankan species would be expected to have their closest relationships. This dense sampling adds confidence to the relationships inferred from our phylogenetic analysis.

The concatenated phylogeny confirms our hypothesis that the Sri Lankan representatives of the genus do not form a monophyletic group. This relationship was hypothesized on the basis of four of the five species (*P. bandula, P. nigrofasciata, P. cumingii* and *P. reval*) exhibiting a similar morphology and being confined to the island’s south-western wet and intermediate zones (rainfall > 2.5 m/y and 1.8–2.5 m/y, respectively). The wet-zone diversifications of several other cypriniform genera have been shown to be monophyletic [e.g., *Systomus*: 11, *Devario*: 14, and *Rasbora*: 15], whereas such diversifications are rare in the dry zone [15, 39]. Of the four species of *Pethia* endemic to the island’s south-western wet zone, only one, *P. reval,* has a range extending into the western intermediate zone, as far north as the Deduru basin. *Pethia melanomaculata*, in contrast, is confined largely to the dry zone, though extending also to the intermediate zone in the east-draining Mahaweli and Gal basins, and the west-draining Deduru basin. It differs from the other four Sri Lankan species of *Pethia* also in morphology (Figs. 3b–c,  8a,  9a). The sister-group relationship of *P. melanomaculata* is not clearly resolved in our phylogenies (Fig. 4). We suspect this species may have a closer phylogenetic relationship to a lineage from peninsular India that is not represented in our dataset. Similar relationships have been observed for other freshwater fishes widespread in the dry zone of Sri Lanka [40–42] with few exceptions [43].

While our results recover Sri Lankan *Pethia* as polyphyletic, the four southwestern wet zone species are not recovered as monophyletic, as hypothesized (Fig. 4). The phylogenetic relationship between *P. nigrofasciata* and *P. bandula* is not clearly resolved. Similarly, the relationship between *P. cumingii* and *P. reval* too, is ambiguous. Further, the sister-group of the clade that includes *P. reval* and *P. cumingii* in all the analyses is a well-supported clade that includes several Indian species. The clade that includes *Pethia nigrofasciata* and *P. bandula* is recovered as the sister group of the clade that includes *P. cumingii*, *P. reval*, *P. ticto*, *P. longicauda* and *P. pookodensis,* with strong node support in all the analyses (Fig. 4, Additional file 1: Figs. S1, S2). Thus, our phylogeny suggests that the five species of Sri Lankan *Pethia* derive from two or three discrete colonization events from the Indian mainland. Multiple colonization events have been recovered also in the case of other freshwater-fish diversifications in Sri Lanka, such as *Laubuka, Rasbora, Devario* and *Systomus* [11, 14, 15, 39]. In those cases, the diversifications in the island’s wet zone within each genus were shown to stem from a single colonization from India. The present findings support two equally parsimonious scenarios for colonization of *Pethia* in Sri Lanka. One is that the common ancestors of *P. melanomaculata*, *P. nigrofasciata* and *P. bandula*, and *P. cumingii* and *P. reval* derive from three independent colonization events from the Indian mainland. If this scenario is confirmed, then *Pethia* would be the first freshwater fish genus in which a wet-zone diversification deriving from multiple independent colonization events has been detected in the island. An alternative scenario would be two colonization events from mainland India, being the common ancestors of *P. melanomaculata* and *P. nigrofasciata*, *P. bandula*, *P. cumingii* and *P. reval*, followed by a back-migration to India.

This is noteworthy because, despite having been connected by a broad isthmus during episodes of low sea level, post-Miocene biotic exchanges of forest-adapted taxa between India and Sri Lanka have been infrequent [5, 10, 11]. Though subaerial for most of the Plio-Pleistocene, the Palk Isthmus appears, because it was too arid, to have acted more of a filter than a conduit for the dispersal of forest-adapted taxa [5, 8, 10]. While *P. nigrofasciata* is a rainforest-adapted species, *P. cumingii* and *P. reval* are not obligatory rainforest associates (discussed below). We hypothesize, based on our results, that the common ancestor of *P. cumingii* and *P. reval* was a generalist. In such a scenario, a back migration to India by the common ancestor of *P. cumingii* and *P. reval* through the arid Palk Isthmus or the colonization of the rainforests of the island’s wet zone, are both plausible.

### *Pethia bandula*—*P. nigrofasciata*

In our *cytb* and concatenated *rag1* + *cytb* trees, *Pethia bandula* renders *P. nigrofasciata* paraphyletic (Fig. 4, Additional file 1: Fig. S2). *Pethia bandula* is a Critically Endangered species confined to a single localized population in a ~ 3-km stretch of a small stream within the Kelani basin [29]. It therefore enjoys strict protection, and sampling is not permitted. As such, we were limited to using the 540–552 bp sequences from the *cytb* locus available on GenBank, much shorter than the 1082 bp contained in the *cytb* sequences newly generated in this study. This may have led to a weakening of the phylogenetic signal represented by *P. bandula*.

It is also possible that *P. bandula* is the result of a recent speciation event. Its range lies at the northern extremity of that of *P. nigrofasciata* (Fig. 1b). If *P. bandula* is an incipient species, it could be that the lineages are as yet incompletely sorted or even introgressed, leading to it and *P. nigrofasciata* not being recovered as reciprocally monophyletic based on the genetic markers used in the present study. The observation of mixed morphology in some populations of *P. nigrofasciata* in the headwaters of the Attanagalu basin proximal to the type locality of *P. bandula* appear consistent with such a hypothesis (Fig. 1g–n, Additional file 1: Table S9). Despite all three molecular species-delimitation methods we applied (PTP, mPTP and ABGD) grouping them as a single species, *P. bandula* and *P. nigrofasciata* are easily distinguished on morphological criteria alone [29]. A genomic approach may reveal clearer structuring and genetic differentiation between the two species, while also revealing evidence of incomplete lineage sorting or hybridization [44–47].

### *Pethia cumingii*—*P. reval*

The phylogenetic relationship between *P. reval* and *P. cumingii* too, is not clearly resolved in our concatenated phylogeny (Fig. 4), with all three molecular species-delimitation methods recovering them as a single species. Nevertheless, *P. reval* and *P. cumingii* are easily distinguished by the red and yellow, respectively, of their fins, in addition to a suite of morphological characters [28]. There is also a clear geographical signal in the phenotypes of the two species (Fig. 2). The red-finned populations occur exclusively in the northern Deduru, Ma, Attanagalu and Kelani basins, whereas yellow-finned populations occur exclusively in the more southerly Bentara and Gin basins. The Kalu basin lies between the northern and southern watersheds that host exclusively the red or yellow-finned populations assigned the names *P. reval* and *P. cumingii*, respectively. While populations in the Kalu usually have yellow fins, individuals with a mix of red and yellow or orange fins occur in some localities (Fig. 2d). It could be that speciation in these two lineages too, is recent, with as yet incomplete lineage sorting or introgression. Both these species are assessed as Endangered [48], and accurate recognition of the species’ taxonomic status is important in conservation management. While our single-locus species-delimitation and the phylogenetic analyses based on *cytb* and *rag1* failed to separate *P. reval* and *P. cumingii*, we adopt a conservative taxonomic approach and retain them as valid species based on their distinct morphology and allopatric distribution. Similar to the case of *P. bandula* and *P. nigrofasciata*, we expect a genome-wide analysis to recover clear structuring and genetic differentiation between these species while also revealing whether the mentioned phenotypic discrepancies in the Kalu basin are the result of hybridization [28, 44–47].

### Geographic ranges and habitats

*Pethia nigrofasciata* is confined to Sri Lanka’s wet-zone basins, from the Attanagalu in the north to the Walawe in the south. It occurs in clear-water streams and rivers with gravel or pebble substrates. The habitats of *P. nigrofasciata* resemble those of the other widespread endemic species in the rainforests of the wet zone, such as *Devario micronema*, *Laubuka varuna*, *Rasbora wilpita* and *Systomus pleurotaenia* [11, 14, 15, 39]. In contrast, *P. reval* and *P. cumingii* occupy broader ecological niches. While the former occurs close to banks in rivers associated with rainforest habitats, it is encountered also in pools in the lowland floodplains and in streams traversing rice paddies, with substrates of silt or debris. Thus, although the extension of its range as far north as the Deduru basin in the intermediate zone is unsurprising, *P. reval* is among the two endemic freshwater fishes that occur in both the wet zone and the intermediate zone, the other such species being the silurid catfish *Ompok argestes* [49].

*Pethia cumingii*, however, is more associated with rainforests, occurring in both shaded streams and rivers. While our samples of this species derive only from the Kalu, Bentara and Gin basins, we have observed it also in the Nilwala and Walawe basins further south. Meanwhile, *Pethia bandula* is confined to a single small stream at Galapitamada, its type locality, which traverses a rice-paddy landscape. This region was likely occupied by rainforest prior to anthropogenic modification. In contrast, compared to its four wet-zone congeners, *P. melanomaculata* occupies a broad ecological niche in the dry and intermediate zones. It occurs in lotic habitats such as rivers, streams, canals, as well as lentic habitats such as seasonal pools and reservoirs and, unlike the other Sri Lankan species of *Pethia*, does not appear to be associated with shade or riparian vegetation.

In some rainforest habitats in the Attanagalu and the Kelani basins, *P. nigrofasciata* and *P. reval* occur in syntopy. Similarly, in such habitats in the Kalu, Bentara, Gin, and Nilwala basins, *P. nigrofasciata* and *P. cumingii* occur in syntopy. While we have not encountered *P. reval* and *P. melanomaculata* in syntopy in the intermediate zone, this would be expected [22, 32]. At the type locality of *P. bandula*, no other species of *Pethia* occurs. However, both *P. nigrofasciata* and *P. reval* occur in nearby streams [22; present study].

### Phylogeography

Several recent studies have explored the phylogeographic structure of cyprinid species confined to Sri Lanka’s southwestern wet zone, such as *Devario micronema, Laubuka varuna, Rasbora wilpita,* and *Systomus pleurotaenia* [11, 14, 15, 39]. All these rainforest associates show strong within-basin genetic structure, with limited gene flow between even adjacent basins. In these cases, it appears that inter-basin dispersal is inhibited by the concerned species being restricted to shaded clearwater streams draining the foothills of the island’s central mountains. They are thus absent from the lowland floodplain, across which there is potentially hydrological connectivity between basins when flooding follows episodes of heavy rainfall. In cases where inter-basin geneflow had in fact occurred, it was inferred that this was the result of headwater river-capture events rather than via the lowland floodplain [5, 11].

*Pethia nigrofasciata* too, shows within-basin phylogeographic structure, with no *cytb* haplotypes shared between basins (Fig. 5). As mentioned above, our concatenated phylogeny (Fig. 4) recovered *P. nigrofasciata* as two well-supported, sympatric subclades, one spanning the distribution of the species in Sri Lanka, from the Attanagalu to the Walawe basins, and the other confined to the region between the Kalu and Gin basins, inclusive. Such a pattern has not been observed in the other phylogeographic studies of Sri Lankan cyprinids published so far [5, 11, 14, 15, 39]. Given that our genetic dataset is limited, it is difficult to offer an explanation for this observation. However, in our *rag1* nuclear dataset (Additional file 1: Fig. S2), these two subclades are not apparent. It is possible that these lineages underwent secondary admixture between allopatrically evolved populations [50]. The complex topography of the southwestern wet zone may have imposed historical biogeographic barriers to gene flow between the two populations. The *cytb* haplotype network of *P. nigrofasciata* too (Fig. 5b), does not suggest inter-basin gene flow through headwater capture between the adjacent basins. The star-like pattern of the *rag1* haplotype network of *P. nigrofasciata* (Fig. 5c), however, may suggest a recent range expansion, even though the neutrality tests were not significant. Broader sampling within each river basin and genome-wide data may reveal a clearer picture of the evolutionary history of these two mitochondrial lineages in *P. nigrofasciata*. While no haplotypes are shared between these two subclades, samples from the same locality may belong to both subclades. For example, the *cytb* haplotypes N10 and N11 occur in members of subclades 1 and 2, respectively, at the same locality in the Bentara basin. The occurrence of distinct mitochondrial lineages in syntopy has also been observed in the southwestern basins of Sri Lanka for the cyprinid *Garra ceylonensis* [5]. This further supports our hypothesis that these samples may have derived from two historically separate matrilineal evolutionary lineages.

While *P. cumingii* and *P. reval* are not obligatory rainforest associates, three of the four subclades of this species-pair exhibit distinct phylogeographic structure (Fig. 4). Subclades A and B contain haplotypes unique to *P. reval,* and subclade D contains haplotypes unique to *P.* cf. *cumingii* from the Kalu basin. Subclade C, however, includes haplotypes shared between both *P. reval* and *P. cumingii*. One *cytb* haplotype, R5, is shared between the adjacent Ma and Attanagalu basins, while another (C5) is shared between the Bentara and Gin. Haplotype C9, however, is disjunct between the Attanagalu (in *P. reval*) and Gin (in *P. cumingii*) basins. It may represent a shared ancestral haplotype which is now fixed in *P. cumingii*. Interestingly, three *cytb* (C1–C3) and three *rag1* (R2–R4) haplotypes are unique to the Kalu basin, that shares no haplotypes with any other basin.

The species of *Pethia* confined to the island’s south-western wet and intermediate zones show evidence of strong phylogeographic structure. Unlike in other endemic cyprinids studied so far, there is little evidence of gene flow between adjacent basins [11, 14, 15, 39]. This could be because the diversification of these lineages has been recent, with ancestral polymorphism retained and lineage sorting as yet incomplete. A genome-wide analysis could provide a clearer understanding of the evolutionary history of these species.

The phylogeographic structure observed in *Pethia melanomaculata* resembles that in *Laubuka lankensis*, which too, has a similar distribution, being confined to the dry and intermediates zones [39]. In both these species, three regional haplogroups can be identified: northwest, Mahaweli, and eastern. Only a single *cytb* haplotype is shared between rivers: the adjacent Kala and Malwathu basins in the northwest haplogroup. While the remaining haplotypes are unique, they are separated by relatively few mutational steps, in contrast to the condition observed in *P. nigrofasciata*, *P. cumingii*, and *P. reval*.

Most of the dry zone’s fishes derive from recent (Pleistocene) dispersants from India, adapted to an arid, strongly seasonal climate [5]. These exhibit little phylogeographic structure [10, 11, 14, 15, 39, 41]. Within the widespread species in the dry zone, the populations from the eastern basins (principally the Gal, Kumbukkan, Menik and Kirindi, which drain the eastern slopes of the central hills) appear to show greater genetic diversity compared with populations in the northwest and the Mahaweli basins. This region lies within the intermediate zone and benefits from higher annual—though less markedly seasonal—rainfall than the northwest and Mahaweli dry zone. It has two endemics confined to it: *Rasbora adisi* and *Laubuka hema* [15, 39]. As *P. melanomaculata* too, demonstrates, the eastern basins show substantial isolation from their neighbours. The region has until now not attracted attention as a focus for conservation, but clearly warrants such consideration.

Nevertheless, perhaps owing to the wet zone’s greater topographic complexity [51], and despite its extent being only about a quarter that of the dry zone, nucleotide and haplotype diversity in the wet-zone endemics *P. nigrofasciata, P. cumingii* and *P. reval* are greater than in *P. melanomaculata*. This phenomenon has been observed previously in species pairs in which one is confined to the wet zone while the other is distributed across the dry zone, such as *Devario micronema* vs *D. malabaricus, Laubuka varuna* vs*. L. lankensis, Systomus pleurotaenia* vs *S. sarana,* and *Rasbora wilpita* vs *R. dandia* [11, 14, 15, 39]. As Potter et al. [52] show, genetic diversity in low-dispersal vertebrate species tends to be higher in mesic, topographically complex biomes, compared to that of species inhabiting dry and topographically less complex biomes.

### Translocations

The haplotype networks of both *P. nigrofasciata* and *P. reval* indicate shared mitochondrial haplotypes (N5 and R8, respectively) between the west-draining Kelani and east-draining Mahaweli basins. These basins share a common boundary along a 40 km long, 600–2000-m high ridge that extends from Ginigathena to the Horton Plains. Sudasinghe et al. [53] reported a shared haplotype between populations of the dwarf snakehead *Channa orientalis* between the two basins, suggesting that gene flow between them is possible. In the case of *Pethia*, however, Wikramanayake [34] recorded a translocation experiment in which both *P. nigrofasciata* and *P. reval* (which he referred to as *Puntius cumingii*) were introduced to a stream near Ginigathena (6.987°N, 80.499°E). Whether stemming from this introduction or other undocumented ones [see: 32], both *P. nigrofasciata* and *P. reval* now occur as far as 40 km downstream, at Peradeniya.

Wikramanayake [34] reported the stocks of *P. reval* and *P. nigrofasciata* introduced to the Mahaweli in 1981 to have come from the Kelani and Kalu basins, respectively. He was not, however, associated with the original translocation experiment and based his report on information from secondary sources. Sudasinghe et al. [33] showed that the stock of *Rasboroides pallidus* translocated in this experiment originated not from the Kalu, as reported by Wikramanayake [34], but likely from the Bentara basin. In the present study, we show the populations of *Pethia* introduced to Mahaweli derive from multiple sources. The Mahaweli populations of *P. reval* contain haplotypes belonging to both subclade A (native to the Deduru, Ma and Attanagalu basins) and subclade B (native to the Kelani). Similarly, the Mahaweli populations of *P. nigrofasciata* contain haplotypes otherwise unique to the Kelani and Kalu basins, in addition to several unique haplotypes, all within the subclade 1 of native *P. nigrofasciata*. In both species, the multiple unique haplotypes in the Mahaweli (R9–R11 in *P. reval,* N20–N23 in *P. nigrofasciata*) suggest that our sampling density underrepresents the haplotype diversity of their native populations.

Deraniyagala [32] reported *P. reval* (as *Puntius cumingii*) from Peradeniya, which suggests that a translocation occurred even before that reported by Wikramanayake [34]. It is also possible that populations of both *P. reval* and *P. nigrofasciata* may have escaped from the fisheries station at Ginigathena, and perhaps also from Peradeniya University, both on the Mahaweli River [54, 55]. The populations of both these fishes in the Mahaweli may thus result from independent founder events spanning several decades, a scenario consistent with our results. This is unsurprising in the light of both species having been popular in the ornamental fish trade for almost a century now.

## Conclusions

Despite *Pethia* being a widespread freshwater fish genus in South Asia, most studies so far have focused on taxonomy, with little or no emphasis on geographic sampling focusing on phylogeographic work. We focus on phylogeny, phylogeography, using nuclear DNA and mitochondrial DNA markers, and compare these results with morphology of the group. Polyphyly in Sri Lankan *Pethia* suggests two or three colonizations from mainland India. Strong phylogeographic structure suggests that the topographically complex wet zone harbors greater genetic diversity than the more uniform dry-zone. Mixed morphological characters between some of the taxa, and their unresolved phylogenies, may suggest recent speciation events with incomplete lineage sorting, or hybridization. The knowledge generated will not only form a foundation for systematics work, but also will help in understanding the processes of speciation and patterns of distribution, allowing for informed conservation of this charismatic group of fishes.

## Methods

### DNA protocols

Gene nomenclature follows ZFIN Zebrafish Nomenclature Conventions (https://goo.gl/MdawKQ). The following new marker sequences were generated for all the Sri Lankan species of *Pethia* except *P. bandula*: 99 mitochondrial *cytochrome b* (*cytb*) and 48 nuclear *recombination activating protein 1* (*rag1*) from 35 locations representative of 14 major river basins in Sri Lanka (Table 1, Figs. 1a, 2a, 3a). Owing to it being Critically Endangered, we lacked permission to obtain fresh samples of *P. bandula.* For this species, therefore, *cytb* sequences based on reliably identified specimens [1, 16, 28] were obtained from GenBank. Methods of DNA extraction, PCR amplification and PCR product purification for *cytb* and *rag1* follow Sudasinghe et al. [4] and Sudasinghe et al. [14], respectively. ChromasPro v1.34 (Technelysium Pty Ltd, Australia) and MEGA v. 7.0 [56] were used to verify the newly generated sequences and to make consensus sequences of the 5′ and 3′ strands, respectively.

The comparative genetic dataset representative of Smiliogastrinae based on Sudasinghe et al. [11], together with additional sequences generated by Katwate et al. [57], Katwate et al. [37], Ren et al. [25] and Sudasinghe et al. [10], were compiled and used in the present study (Additional file 1: Table S2). Among the 16 valid species of *Pethia* from Sri Lanka and peninsular India, 13 are represented in our *cytb* dataset, based on reliably identified specimens [present study; 16, 19, 23, 24, 28, 30, 31, 58]. This reference dataset thus allowed us to confidently identify some incorrectly identified or dubious GenBank sequences. The only three species of *Pethia* from the Indian peninsula not represented in our dataset are *P. narayani*, *P. sharmai* and *P. striata*. Of these, the generic placement of the first two is doubtful [see Discussion; also 16,19]. Unfortunately, we lack a nuclear (*rag1*) reference dataset, based on reliably identified specimens, of Indian *Pethia*. The available *rag1* sequences in GenBank are in any case scarce compared with *cytb*. Some of the available *rag1* sequences, however, are accompanied by *cytb* sequences derived from the same voucher specimens [e.g., 25]. This allowed us to infer the identification of these *rag1* sequences from their *cytb* counterparts. In cases where the GenBank identification is doubtful, however, we place the species name within double quotes.

The *cytb* and *rag1* sequences were aligned independently, using ClustalW in MEGA v. 7.0 [56] and each alignment checked and translated to verify the absence of frameshift mutations and premature stop codons. The online program FaBox [59] was used to condense the sequences into unique haplotypes. PhyloSuite v1.2.1 was used in data concatenation and conversion of sequence formats [60].

### Phylogenetic analysis

For each single gene dataset of *cytb* (1082 bp, 395 taxa) and *rag1* (1490 bp, 204 taxa), and for the concatenated dataset of *cytb* + *rag1* (2572 bp, 371 taxa), phylogenetic inferences based on Maximum Likelihood (ML) and Bayesian inference (BI) were made using RAxML-NG [61] and MrBayes v3.2 [62] through the CIPRES Science Gateway [63].

The optimal nucleotide substitution model and partitioning schemes for the BI analysis were evaluated using PartitionFinder 2 [64] through the CIPRES Science Gateway. Each codon position of each gene was given as the starting subset, branch lengths as “linked”, model as MrBayes, model selection under the Bayesian information criterion (BIC), and search method as the “greedy” algorithm [65]. These were then evaluated using PhyML 3.0 [66] in the PartitionFinder 2 package. Four Metropolis coupled Markov-chain Monte Carlo (MCMCMC) chains in two independent runs of 10 million generations, with a sampling interval of 1000 each, were conducted in MrBayes v3.2 for the BI analysis. Convergence of the two runs was checked using Tracer [67] and the burn-in fraction set as 0.1. Statistical support for the nodes in the BI analyses was determined using the trees which remained after burn-in, based on the Bayesian posterior probabilities (PP) of the clades [68].

ModelTest-NG [69] through the CIPRES Science Gateway was used to determine the optimal nucleotide substitution model for the ML inference, using the minimum AIC score. Statistical support for the nodes in the ML inference was determined by Felsenstein’s bootstrap method for 1000 replicates. The trees obtained from BI and ML analyses were visualized using Figtree v1.4.3 (http://tree.bio.ed.ac.uk/software/figtree). The different partitioning schemes, evolutionary models and number of sequences used in the analyses are provided in Additional file 1: Table S3.

### Molecular species delimitation

Molecular species delimitation for single-locus data includes several tree-based and distance-based methods [70–75]. Use of a combination of these approaches is increasingly applied to overcome the weaknesses of individual methods [76–79]. We used three different single-locus molecular species-delimitation algorithms to infer the species boundaries in our dataset: the distance-based Automatic Barcode Gap Discovery (ABGD) [73], the tree-based multi-rate Poisson Tree Processes (mPTP) [71], and the Poisson Tree Processes (PTP) [75].

The ABGD analysis was run using the Unix command-line version available from https://bioinfo.mnhn.fr/abi/public/abgd/. The entire dataset of *cytb* for *Pethia* was run in ABGD under the JC69, K80 and uncorrected p-distance models while varying the minimum gap width (X) as X = 1.5, X = 1.0 and X = 0.8. The rest of the settings were retained at their default values.

The tree-based species-delimitation methods were applied to a 145-taxon *cytb* dataset after condensing it into unique haplotypes. The Unix command-line software mptp 0.2.4 [71] was used to conduct the mPTP and PTP analyses. The options ‘-multi’ and ‘-single’ in the mptp software were chosen to decide the algorithm for the mPTP and PTP analyses, respectively. A ML gene tree generated by RAxML-NG was used as the starting binary tree. The MCMC analyses for both mPTP and PTP were run for 50 million generations, sampling every 10,000 generations, in two independent runs after removing the outgroups. The first 1,000,000 trees were discarded as burn-in and the convergence of the two runs checked by the plot of generation vs. log-likelihood.

In addition, we also calculated, using MEGA, the uncorrected pairwise *cytb* genetic distances for species of *Pethia*.

### Genetic diversity and population structure

For *cytb* and *rag1*, we estimated genetic diversity within *P. cumingii*, *P. melanomaculata*, *P. nigrofasciata*, and *P. reval* by computing the number of haplotypes (h), polymorphic sites (S), parsimony-informative sites (P), nucleotide diversities (π) and haplotype diversities (Hd) using DNAsp v.6 [80]. The neutrality tests, Tajima’s D [81] and Fu and Li's F [82], were conducted using DNAsp v.6 to explore demographic changes in the above-mentioned four species of *Pethia*. The haplotype networks for *cytb* and *rag1* for these four species were constructed through a Median-Joining Network [83] in PopArt [84].

### Morphometry, meristics and statistical analysis

Metric and meristic data were obtained from the examination of a total of 386 and 380 specimens, respectively, following the methods of Sudasinghe et al. [4]. All bilateral measurements were taken point-to-point on the left side of specimens using a digital caliper to the nearest 0.1 mm. The number in parentheses after a count indicates the frequency of that count. Specimens examined (Additional file 1: Table S4) are deposited in the collection of the Wildlife Heritage Trust of Sri Lanka (WHT), now at the National Museum of Sri Lanka, Colombo, Sri Lanka (NH); the Evolutionary Ecology and Systematics Lab, Department of Molecular Biology and Biotechnology, University of Peradeniya, Peradeniya, Sri Lanka (DZ); the collection of Maurice Kottelat, Delémont, Switzerland (CMK); and the Zoological Reference Collection, Lee Kong Chian Natural History Museum, National University of Singapore, Singapore (ZRC).

All the measurements showed a positive correlation with the standard length. Therefore, size correction for the measurements was done using the equation,

$$M_{s} = M_{o} \left( {\frac{{L_{s} }}{{L_{o} }}} \right)^{b}$$

where the standardized measurement and the measured character length are represented by $M_{s}$ and $M_{o}$, respectively, $L_{o}$ is the standard length of each specimen, and $L_{s}$ is the overall (arithmetic) mean standard length for all individuals from all populations of all the species. The value of $b$ for each character from the observed data was estimated using the allometric-growth equation $M = aL^{b}$, where $b$ is the gradient of regression of $\log M_{o}$ on $\log L_{o}$ [85].

Principal component analyses (PCA) using a correlation matrix were carried out to visualize and summarize multivariate morphometric data in a few dimensions and to assess whether the different species of *Pethia* and geographic subgroups form distinct clusters. A multiple correspondence analysis (MCA) was carried out to visualize and summarize the most variable meristic data (scale counts) in a few dimensions and to assess whether the different species and subgroups of *Pethia* form distinct clusters. The fin-ray counts were excluded from the MCA as they were invariable among all the species examined. All statistical analyses were done using R Studio 4.0.0 [86] and the R package FactoMineR (version 1.34) was used for the PCA and MCA [87].

## Supplementary Information

**Additional file 1: Fig. S1.** Molecular phylogenetic relationships of *Pethia*, based on Bayesian inference of the *cytb* (1082 bp) data set. Asterisks (*) above and below nodes represent ≥ 95% Bayesian posterior probabilities and ML bootstrap values, respectively. Scale bar represents number of changes per site. Node support below 50 is not labeled. **Fig. S2.** Molecular phylogenetic relationships of *Pethia*, based on Bayesian inference of the *rag1* (1490 bp) data set. Asterisks (*) above and below nodes represent ≥ 95% Bayesian posterior probabilities and ML bootstrap values, respectively. Scale bar represents number of changes per site. Node support below 50 is not labeled. **Table S1.** Valid species of *Pethia*, with their type localities, distinguishing characters and distribution. **Table S2.** The comparative genetic dataset representative of Smiliogastrinae and outgroups downloaded from GenBank. **Table S3.** Nucleotide substitution models and the partitions used in the phylogenetic analyses. **Table S4.** Specimens of *Pethia* examined for the morphological analysis. LK, Sri Lanka; IND, India. **Table S5**. Intraspecific uncorrected pairwise *cytb* genetic distances for species of *Pethia* in Sri Lanka. **Table S6.** Genetic diversity, based on *cytb* and *rag1*, in Sri Lankan species of *Pethia*. Number of sequences (N), number of haplotypes (h), polymorphic sites (S), parsimony-informative sites (P), nucleotide diversity (π), haplotype diversity (Hd). None of the neutrality tests were statistically significant. **Table S7.** Component loadings in the principal component analysis of the size-adjusted morphometric measurements of species of *Pethia* in Sri Lanka. **Table S8.** Proportional morphometric data for the species of *Pethia* in Sri Lanka. **Table S9.** Frequency distribution of selected meristic data in the Sri Lankan species of *Pethia* examined in the present study. **Table S10.** Proportional morphometric data for *Pethia bandula*, *P. nigrofasciata* (excluding Attanagalu populations), and *P.* cf. *nigrofasciata* (Attanagalu population). **Table S11.** Proportional morphometric data for *Pethia reval*, *P. cumingii* (Bentara and Gin populations), and *P.* cf. *cumingii* (Kalu population) in Sri Lanka.

## Data Availability

All data generated or analyzed during this study or the sources of data (GenBank) are included in this published article.

## Abbreviations

ABGD: Automatic Barcode Gap Discovery
a.s.l: Above sea level
BI: Bayesian Inference
BIC: Bayesian information criterion
BP: Felsenstein’s bootstrap support
bp: Base pairs
*cytb*: *cytochrome b*
kya: Thousand years ago
MCA: Multiple correspondence analysis
MCMCMC: Metropolis coupled Markov-chain Monte Carlo
ML: Maximum Likelihood
mPTP: Multi-rate Poisson Tree Processes
My: Million years ago
m/y: Meters per year
PCA: Principal component analysis
PCR: Polymerase chain reaction
PP: Bayesian posterior probability
PTP: Poisson Tree Processes
*rag1*: *recombination activating gene 1*
SL: Standard length
s.s: Sensu stricto
