# Supplementary material for: Molecular phylogeny and phylogeography of the freshwater-fish genus Pethia (Teleostei: Cyprinidae) in Sri Lanka
Source: BMC Ecol Evol. 2021 Nov 10;21:203. doi: 10.1186/s12862-021-01923-5 (PMC8582130; doi:10.1186/s12862-021-01923-5)
Supplement: Supplementary file 1 — Additional file 1: Fig. S1. Molecular phylogenetic relationships of Pethia, based on Bayesian inference of the cytb (1082 bp) data set. Asterisks (*) above and below nodes represent ≥ 95% Bayesian posterior probabilities and ML bootstrap values, respectively. Scale bar represents number of changes per site. Node support below 50 is not labeled. Fig. S2. Molecular phylogenetic relationships of Pethia, based on Bayesian inference of the rag1 (1490 bp) data set. Asterisks (*) above and below nodes represent ≥ 95% Bayesian posterior probabilities and ML bootstrap values, respectively. Scale bar represents number of changes per site. Node support below 50 is not labeled. Table S1. Valid species of Pethia, with their type localities, distinguishing characters and distribution. Table S2. The comparative genetic dataset representative of Smiliogastrinae and outgroups downloaded from GenBank. Table S3. Nucleotide substitution models and the partitions used in the phylogenetic analyses. Table S4. Specimens of Pethia examined for the morphological analysis. LK, Sri Lanka; IND, India. Table S5. Intraspecific uncorrected pairwise cytb genetic distances for species of Pethia in Sri Lanka. Table S6. Genetic diversity, based on cytb and rag1, in Sri Lankan species of Pethia. Number of sequences (N), number of haplotypes (h), polymorphic sites (S), parsimony-informative sites (P), nucleotide diversity (π), haplotype diversity (Hd). None of the neutrality tests were statistically significant. Table S7. Component loadings in the principal component analysis of the size-adjusted morphometric measurements of species of Pethia in Sri Lanka. Table S8. Proportional morphometric data for the species of Pethia in Sri Lanka. Table S9. Frequency distribution of selected meristic data in the Sri Lankan species of Pethia examined in the present study. Table S10. Proportional morphometric data for Pethia bandula, P. nigrofasciata (excluding Attanagalu populations), and P. cf. nigrofasciata (Attan [file 12862_2021_1923_MOESM1_ESM.pdf]

Additional Information for

Molecular phylogeny and phylogeography of the freshwater-fish genus *Pethia* (Teleostei: Cyprinidae) in Sri Lanka

This includes:

Figures S1 to S2

Tables S1 to S11

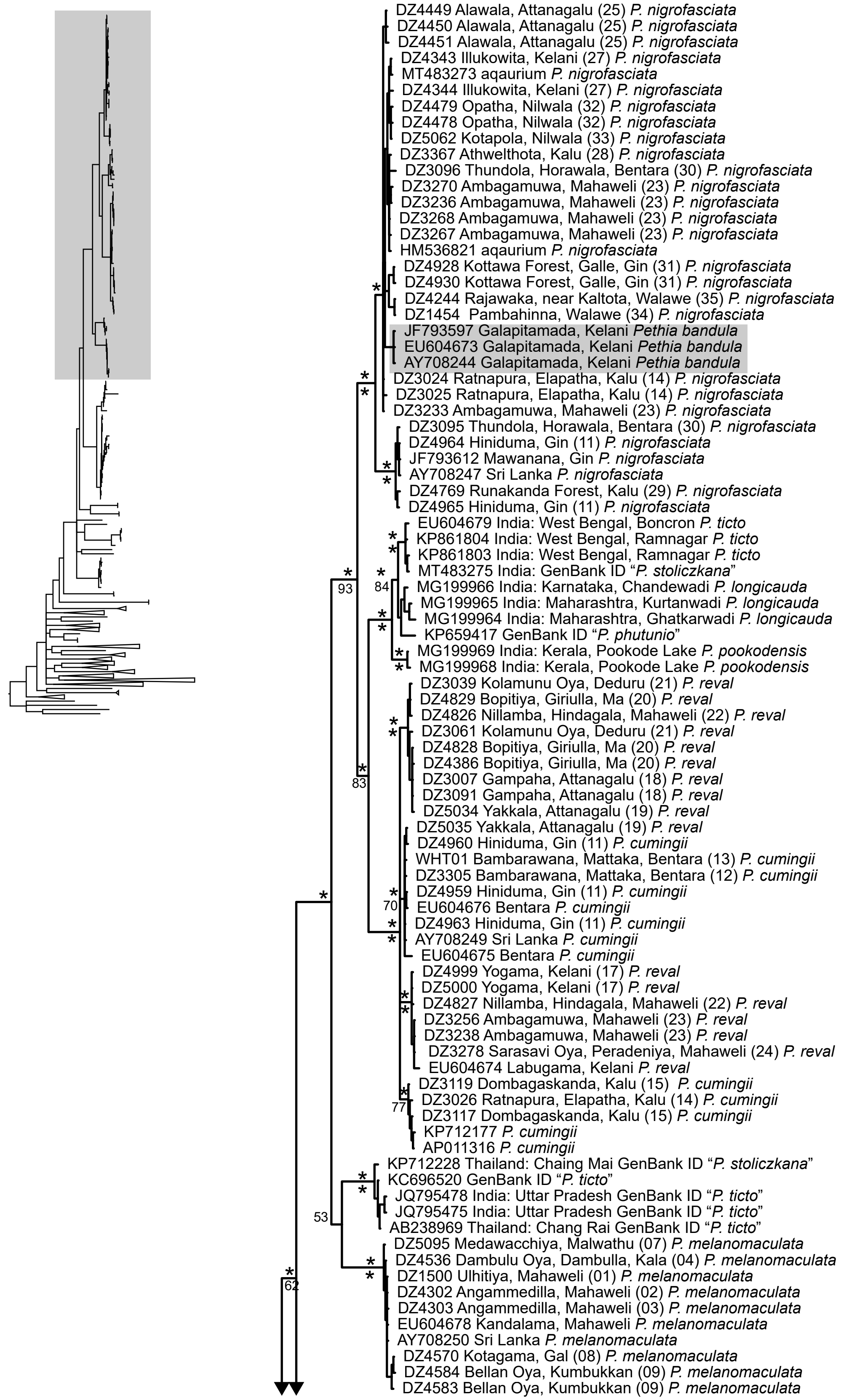

Fig. S1. Molecular phylogenetic relationships of *Pethia*, based on Bayesian inference of the cytb (1082 bp) data set. Asterisks (*) above and below nodes represent ≥ 95% Bayesian posterior probabilities and ML bootstrap values, respectively. Scale bar represents number of changes per site. Node support below 50 is not labeled.

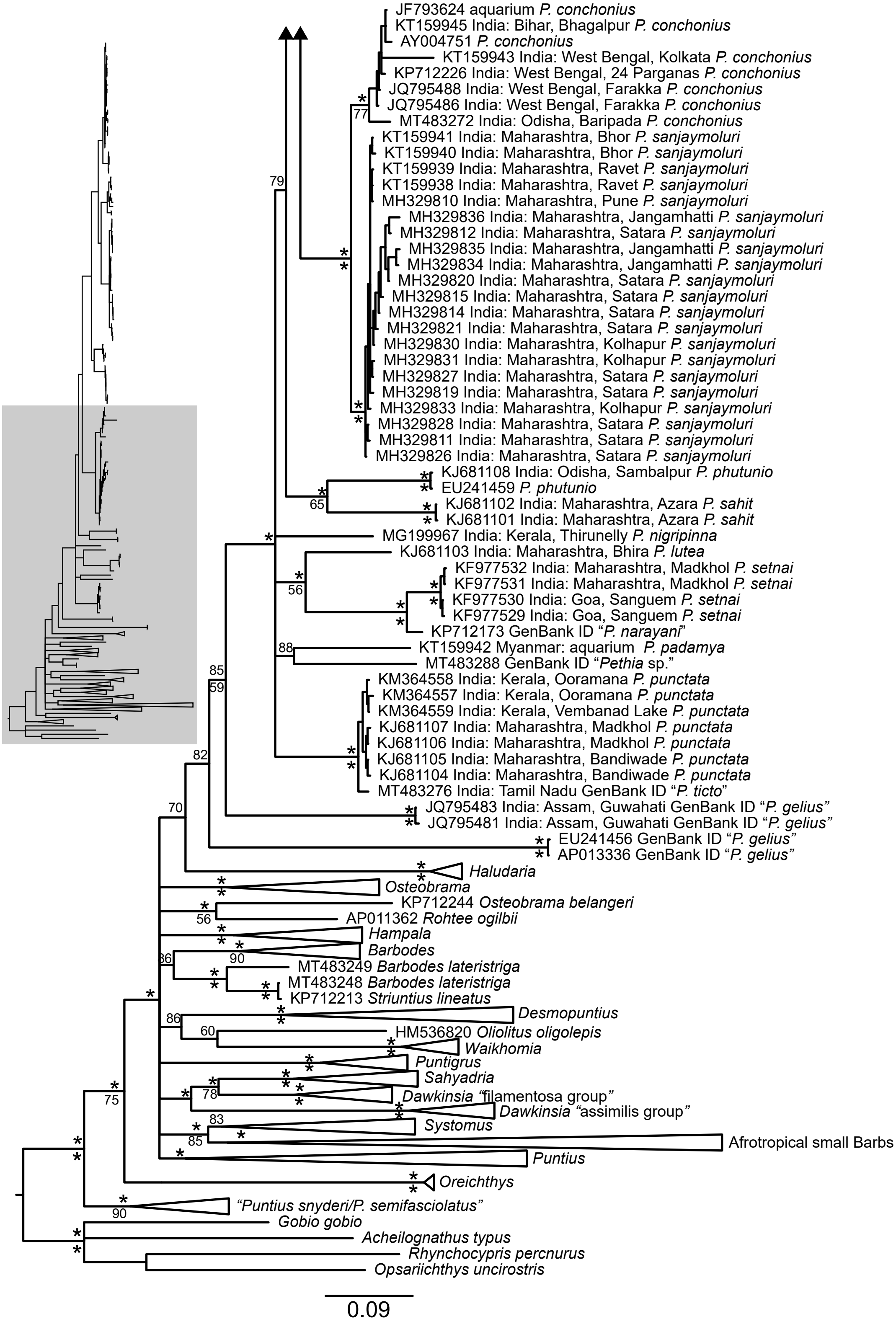

Fig. S1 Contd. Molecular phylogenetic relationships of *Pethia*, based on Bayesian inference of the cytb (1082 bp) data set. Asterisks (*) above and below nodes represent ≥ 95% Bayesian posterior probabilities and ML bootstrap values, respectively. Scale bar represents number of changes per site. Node support below 50 is not labeled.

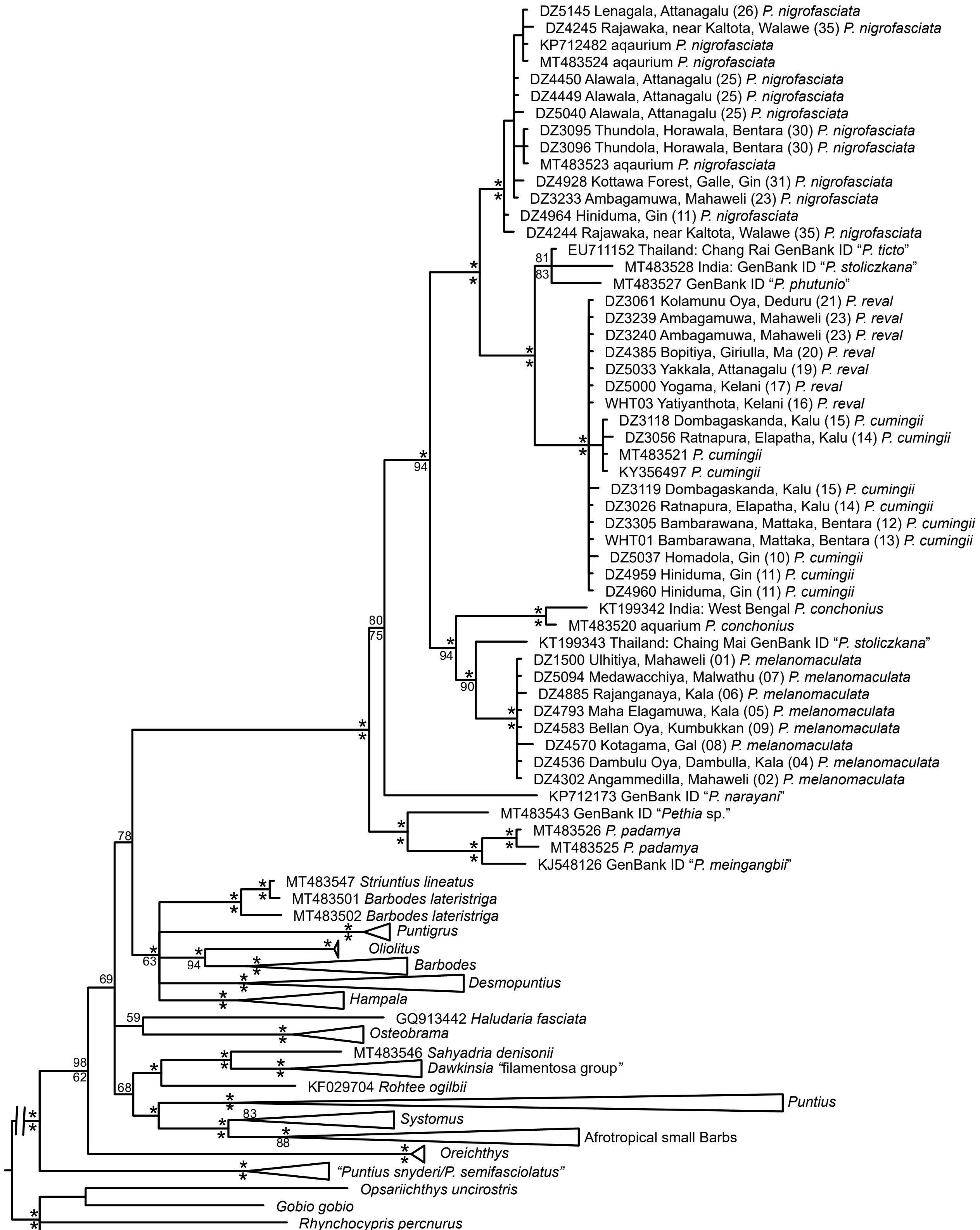

Fig. S2. Molecular phylogenetic relationships of *Pethia*, based on Bayesian inference of the *rag1* (1490 bp) data set. Asterisks (*) above and below nodes represent ≥ 95% Bayesian posterior probabilities and ML bootstrap values, respectively. Scale bar represents number of changes per site. Node support below 50 is not labeled.

Table S1. Valid species of *Pethia*, with their type localities, distinguishing characters and distribution.

Species	Author & year	Type locality	Distribution	Characters	Validated by
***Pethia* sensu stricto**					
Pethia arunachalensis	Shangningam, Kosygin & Chowdhury 2020	India: Arunachal Pradesh, Changlang District, Nao-dhing River near Miao, Brahmaputra drainage, 27.5044 N, 96.1808 E	Northeast India: Arunachal Pradesh, Changlang District, Nao-dhing River (Brahmaputra drainage)	Lateral line incomplete with 6-7 pored, 22-23 scales along lateral row; 4.5 scales between dorsal-fin origin and lateral line; barbels absent; caudal blotch rounded; humeral blotch absent	Shangningam et al. (2020)
P. atra	(Linthoingambi & Vishwanath 2007)	India: Manipur, Iril River at Bamon-Kampu	Northeast India: Manipur, Iril and Imphal drainages	Lateral line incomplete with 5-11 pored, 25-29 scales along lateral row; 4.5 scales between dorsal-fin origin and lateral line; barbels absent; caudal blotch small, rounded; humeral blotch absent	Linthoingambi & Vishwanath (2007)
P. bandula	(Kottelat & Pethiyagoda 1991)	Sri Lanka: Near Galapitamada, Kelani drainage, 7.16 N, 80.25 E	Sri Lanka: Near Galapitamada, Kelani drainage	Lateral line incomplete with 8-11 pored, 19-21 scales along lateral row; 4.5 scales between dorsal-fin origin and lateral line; barbels absent; caudal blotch elongated as a bar; humeral blotch vertically elongated as a bar	Kottelat & Pethiyagoda (1991); Meegaskumbura et al. (2008); Pethiyagoda et al. (2012)
P. conchonius	(Hamilton 1822)	India: Assam, at Gualpara [Goalpara], Ponds near Brahmaputra River	Northeast India: Brahmaputra drainage	Lateral line incomplete with 7-17 pored, 24-26 scales along lateral row; 5.5 scales between dorsal-fin origin and lateral line; barbels absent; caudal blotch rounded; humeral blotch absent	Kullander & Fang (2005); Kullander (2008); Pethiyagoda et al. (2012); Kottelat (2013); Katwate et al. (2016); Shangningam & Vishwanath (2018)
P. cumingii	(Günther 1868)	Sri Lanka [Ceylon]	Sri Lanka: Kalu, Bentara, Gin and Nilwala drainages in southwestern wet zone of Sri Lanka	Lateral line incomplete with 4-8 pored, 17-20 scales along lateral row; 3-3.5 scales between dorsal-fin origin and lateral line; barbels absent; caudal blotch elongated as a bar; humeral blotch elongated as a bar	Günther (1868); Meegaskumbura et al. (2008); Pethiyagoda et al. (2012); Batuwita et al. (2015); present study

P. didi	(Kullander & Fang 2005)	Myanmar: Kachin State, Hpa Lap Chaung just south of Yuzana Myiang village, Ayeyarwaddy drainage, 25.5236 N, 97.3719 E	Myanmar: Kachin State, Myitkyina and Lake Indawgyi, Ayeyarwaddy drainage	Lateral line incomplete with 6-7 (rarely 10) pored, 19-21 scales along lateral row; 4.5 scales between dorsal-fin origin and lateral line; maxillary barbels present, rudimentary; caudal blotch elongated as a bar; humeral blotch absent	Kullander & Fang (2005)
P. erythromycter	(Kullander 2008)	Myanmar: Kachin State, Hap Lap Chaung just south of Yuzana Myaing village, Ayeyarwaddy drainage	Myanmar: Kachin State, Myitkyina and Lake Indawgyi, Ayeyarwaddy drainage	Lateral line incomplete with 4-7 pored, 18-20 scales along lateral row; 4.5 scales between dorsal-fin origin and lateral line; barbels absent; caudal blotch elongated as a bar; humeral blotch absent	Kullander (2008)
P. expletiforis	Mayanglambam & Vishwanath 2013	India: Mizoram, Saiha District, Ka-ao River near New Serkawr village, Kaladan drainage, 22.3527 N, 92.9636 E	Northeast India: Mizoram, Saiha District, Ka-ao River, Kaladan drainage	Lateral line complete with 21-23 pored scales; 4.5 scales between dorsal-fin origin and lateral line; barbels absent; caudal blotch rounded; humeral blotch absent	Mayanglambam & Vishwanath (2013)
P. khugae	(Linthoingambi & Vishwanath 2007)	India: Manipur, Khugae River, at Churachandpur district, Chindwin drainage	Northeast India: Manipur, Khugae River	Lateral line incomplete with 8-11 pored, 28-30 scales along lateral row; 5.5 scales between dorsal-fin origin and lateral line; barbels absent; caudal blotch small, rounded; humeral blotch absent	Linthoingambi & Vishwanath (2007)
P. longicauda	Katwate, Paingankar, Raghavan & Dahanukar 2014	India: Maharashtra, Kolhapur District, Gavse-Ajara, Hiranyakeshi River, 16.0683 N, 74.0916 E	South India: Western Ghats, Hiranyakeshi drainage	Lateral line incomplete with 5-6 pored, 22-24 scales along lateral row; 3.5 scales between dorsal-fin origin and lateral line; barbels absent; 2 caudal blotches: 1st one distinct, rounded, 2nd one smaller and hazy; humeral blotch present, small, rounded, on 3rd and 4th lateral-line scale and extending one scale above	Katwate et al. (2014a)
P. lutea	Katwate, Katwate, Raghavan, Paingankar & Dahanukar 2014	India: Maharashtra, Raigad District, Bhira, Kundalika River, 18.4410 N, 73.2670 E	South India: Western Ghats, Maharashtra, west-flowing drainages between 17-19° latitudes	Lateral line complete with 18-21 pored scales; 4-4.5 scales between dorsal-fin origin and lateral line; barbels absent; caudal blotch rounded; humeral blotch present, on 3rd and 4th lateral-line scale and extending one scale above and below as a bar	Katwate et al. (2014b)
P. macrogramma	(Kullander 2008)	Myanmar: Kachin State, Nan Kywe Chaung, Ayeyarwaddy drainage	Myanmar: Kachin State, streams near Myitkyina, Ayeyarwaddy drainage	Lateral line complete (or almost complete or interrupted) with 19-23 pored, 21-23 scales along lateral row; 4.5 scales between dorsal-fin origin and lateral line; barbels absent; caudal	Kullander (2008)

				blotch rounded; humeral blotch present, small, rounded	
P. manipurensis	(Menon, Rema Devi & Vishwanath 2000)	India: Manipur, Moirang, Lake Loktak, Chindwin drainage	Northeast India: Manipur, Moirang, Lake Loktak, Chindwin drainage	Lateral line incomplete with 5-6 pored, 22-24 scales along lateral row; 3.5 scales between dorsal-fin origin and lateral line; barbels absent; caudal blotch small, rounded; humeral blotch present, small, rounded, on 3rd and 4th lateral-line scale	Menon et al. (2000)
P. meingangbii	(Arunkumar & Tombi Singh 2002)	India: Manipur, Moreh Bazar, Moreh 110 kilometers from Imphal, Chindwin drainage	Northeast India: Manipur, Chindwin drainage	Lateral line incomplete with 6 pored, 21-23 scales along lateral row; 5.5 scales between dorsal-fin origin and lateral line; barbels absent; caudal blotch rounded; humeral blotch present, small, rounded, on 3rd and 4th lateral-line scale	Arunkumar et al. (2002)
P. melanomaculata	(Deraniyagala 1956)	Sri Lanka: Kantale reservoir, Mahaweli drainage	Sri Lanka: drainages in the dry zone	Lateral line incomplete with 5-21 pored, 18-22 scales along lateral row; 4-4.5 scales between dorsal-fin origin and lateral line; barbels absent; caudal blotch small, rounded; humeral blotch present, small, rounded, on 3rd and 4th lateral-line scale	Batuwita et al. (2015); present study
P. nankyweensis	(Kullander 2008)	Myanmar: Kachin State, Nan Kywe Chaung, Ayeyarwaddy drainage	Myanmar: Kachin State, streams near Myitkyina, Ayeyarwaddy drainage	Lateral line incomplete with 5-6 pored, 20-23 scales along lateral row; 3.5 scales between dorsal-fin origin and lateral line; maxillary barbels present; caudal blotch elongated as a bar; humeral blotch absent	Kullander (2008)
P. nigripinnis	(Knight, Rema Devi, Indra & Arunachalam 2012)	India: Kerala, Wyanad, Kalindhi stream, Kabini drainage, 11.78 N, 76.06 E	South India: Western Ghats, Moyar and Kabini drainages	Lateral line incomplete with 3-5 pored, 20-21 scales along lateral row; 4.5 scales between dorsal-fin origin and lateral line; barbels absent; caudal blotch elongated as a bar; humeral blotch present, small, rounded, on 3rd and 4th lateral-line scale	Knight et al. (2012)
P. nigrofasciata	(Günther 1868)	Southern Sri Lanka [Ceylon]	Sri Lanka: Attanagalu, Kelani, Kalu, Bentara, Gin, Nilwala and Walawe drainages in southwestern wet zone of Sri Lanka; translocated populations in Mahaweli drainage of Central Hills	Lateral line complete with 17-22 pored scales; 17-22 scales between dorsal-fin origin and lateral line; barbels absent; caudal blotch elongated as a bar; humeral blotch elongated as a bar; a third bar beneath the base of dorsal fin	Günther (1868); Meegaskumbura et al. (2008); Pethiyagoda et al. (2012); Batuwita et

					al. (2015); present study
P. ornata	(Vishwanath & Laisram 2004)	India: Manipur, Moreh, Lokchao River	Northeast India: Manipur, Lokchao River, Chindwin drainage	Lateral line incomplete or interrupted with 5-22 pored scales, 20-25 scales along lateral row; 4.5 scales between dorsal-fin origin and lateral line; barbels absent; caudal blotch elongated as a bar; humeral blotch absent	Vishwanath & Laisram (2004);
P. padamya	(Kullander & Britz 2008)	Myanmar: Mandalay Division, Toe Gyi village, above Aniskan falls, Ayeyarwaddy drainage, 21.976 N, 96.390 E	Myanmar: Mandalay Division, Chindwin drainage	Lateral line incomplete with 5-8 pored scales, 19-21 scales along lateral row; 4.5 scales between dorsal-fin origin and lateral line; maxillary barbels present (rudimentary); caudal blotch small, rounded; humeral blotch elongated as a bar	Kullander & Britz (2008)
P. phutunio	(Hamilton 1822)	India: northeastern Bengal, Ponds at Pergunj	Northeast India: Ganga-Brahmaputra drainages	Lateral line incomplete with 3-5 pored scales, 20-23 scales along lateral row; 3.5 scales between dorsal-fin origin and lateral line; maxillary barbels present (rudimentary); caudal blotch rounded; humeral blotch elongated as a bar; a third bar beneath the base of dorsal fin	Menon et al. (2000); Kullander & Fang (2005); Pethiyagoda et al. (2012); Kottelat (2013); Katwate et al. (2018)
P. poiensis	Shangningam & Vishwanath 2018	India: Manipur, Ukhrul District, Challou River at Poi Village, Chindwin drainage, 25.28 N, 94.51 E	Northeast India: Manipur, Ukhrul District, Challou River, Chindwin drainage	Lateral line incomplete with 7-9 pored scales, 19-20 scales along lateral row; 3.5 scales between dorsal-fin origin and lateral line; barbels absent; caudal blotch small, rounded; humeral blotch small, rounded, on 3rd and 4th lateral-line scale	Shangningam & Vishwanath (2018)
P. pookodensis	(Mercy & Jacob 2007)	India: Kerala, Wyanad, Pookode Lake	South India: Kerala, Western Ghats, Wyanad, Pookode Lake	Lateral line incomplete with 6-8 pored scales, 22-23 scales along lateral row; 3.5 scales between dorsal-fin origin and lateral line; barbels absent; caudal blotch small, rounded; humeral blotch small, rounded, on 3rd and 4th lateral-line scale	Mercy & Jacob (2007); Katwate et al. (2018)
P. punctata	(Day 1865)	India: Kerala, Cochin	South India: Western Ghats, mostly confined to west-flowing drainages between 8-16° latitudes	Lateral line complete with 23-25 pored scales; 4.5 scales between dorsal-fin origin and lateral line; barbels absent; caudal blotch small, rounded; humeral blotch small, rounded, on 4th scale below the lateral-line row, two-minute spots below the humeral blotch	Katwate et al. (2014c)

P. reval	(Meegaskumbura, Silva, Maduwage & Pethiyagoda 2008)	Sri Lanka: Labugama, Kelani basin, 6.85 N, 80.16 E	Sri Lanka: Attanagalu, and Kelani drainages in southwestern wet zone; Ma and Deduru drainages in northwest intermediate zone of Sri Lanka; translocated populations in Mahaweli drainage of Central Hills	Lateral line incomplete with 4-7 pored, 17-21 scales along lateral row; 3.5 scales between dorsal-fin origin and lateral line; barbels absent; caudal blotch elongated as a bar; humeral blotch elongated as a bar	Meegaskumbura et al. (2008); Pethiyagoda et al. (2012); Batuwita et al. (2015); present study
P. rutila	Lalramliana, Knight & Laltlanhlua 2014	India: Mizoram, Phuldungsei Village, Aivapui River, 23.474 N, 92.390 E	Northeast India: Mizoram, Karnaphuli drainage	Lateral line complete with 21-22 pored scales; 4.5 scales between dorsal-fin origin and lateral line; barbels absent; caudal blotch rounded; humeral blotch small, rounded, on 3rd and 4th scales below the lateral-line row	Lalramliana et al. (2014)
P. sahit	Katwate, Kumkar, Raghavan & Dahanukar 2018	India: Maharashtra, Kolhapur District, Ajara Taluk, near Ghatkarwadi, Hiranyakeshi River, 16.054 N, 74.066 E	South India: Western Ghats, Hiranyakeshi drainage	Lateral line incomplete with 3-6 pored scales, 19-22 scales along lateral row; 4.5 scales between dorsal-fin origin and lateral line; barbels absent; caudal blotch small, rounded; humeral blotch on 3rd and 4th lateral-line scale and extending one scale above and below	Katwate et al. (2018)
P. sanjaymoluri	Katwate, Jadhav, Kumkar, Raghavan & Dahanukar 2016	India: Maharashtra, Rawet, Pavana River, 18.641 N, 73.753 E	South India: Western Ghats, Bhima drainage	Lateral line incomplete with 7-12 pored scales, 23-25 scales along lateral row; 4.5 scales between dorsal-fin origin and lateral line; barbels absent; caudal blotch small, rounded; humeral blotch on 3rd and 4th lateral-line scale and extending one scale below	Katwate et al. (2016)
P. setnai	(Chhapgar & Sane 1992)	India: Goa, Sanguem	South India: Western Ghats, Goa	Lateral line complete with 19-21 scales along lateral row; 3.5-4.5 scales between dorsal-fin origin and lateral line; barbels absent; caudal blotch elongated as a bar; humeral blotch elongated as a bar; a third bar beneath the base of dorsal fin	Katwate et al. (2014c)
P. shalynius	(Yazdani & Talukdar 1975)	India: Meghalaya, about 20 kilometers north of Shillong, Khasi Hills, Barapani Lake	Northeast India: Ganga-Brahmaputra drainages	Lateral line incomplete with 11 pored scales, 20-23 scales along lateral row; 3-4 scales between dorsal-fin origin and lateral line; barbels absent; 2 caudal blotches; humeral blotch absent	Yazdani & Talukdar (1975); Shangningam & Vishwanath (2018)
P. stoliczkana	(Day 1871)	Eastern Myanmar	Northeast India: Chindwin-Irrawaddy drainages	Lateral line complete with 19-23 pored, 21-24 scales along lateral row; 5.5 scales between dorsal-fin origin and lateral line; barbels absent;	Kullander & Fang (2005); Linthoingambi &

				caudal blotch rounded; humeral blotch vertically elongated as a bar	Vishwanath (2007); Kullander (2008); Kottelat (2013)
P. striata	Atkore, Knight, Rema Devi & Krishnaswamy 2015	India: Karnataka, Chikmagalur District, Balipehalla, Mudba stream, Tunga drainage	South India: Western Ghats, Tunga drainage	Lateral line complete with 20-21 scales along lateral row; 4.5 scales between dorsal-fin origin and lateral line; barbels absent; caudal blotch rounded; humeral blotch on 4th scale, below the lateral-line row	Atkore et al. (2015)
P. thelys	(Kullander 2008)	Myanmar: Kachin State, stream about 24 kilometers on road Myitkyina-Myitzon, Ayeyarwaddy drainage	Myanmar: Kachin State, Myitkyina and Lake Indawgyi, Ayeyarwaddy drainage	Lateral line incomplete with 6-11 pored, 20-23 scales along lateral row; 4.5 scales between dorsal-fin origin and lateral line; barbels absent; caudal blotch elongated as a bar; humeral blotch absent	Kullander (2008)
P. tiantian	(Kullander & Fang 2005)	Myanmar: Kachin State, Nan Hto Chaung, in Putao, Ayeyarwaddy drainage, 27.3288 N, 97.3766 E	Myanmar: Kachin State, in Putao, Ayeyarwaddy drainage	Lateral line complete with, 19-21 scales along lateral row; 4.5 scales between dorsal-fin origin and lateral line; barbels absent, rudimentary (absent in some); caudal blotch rounded; humeral blotch elongated as a bar	Kullander & Fang (2005)
P. ticto	(Hamilton 1822)	South eastern parts of Bengal	Northeast India: Ganga-Brahmaputra drainages	Lateral line incomplete with 6-12 pored, 23-26 scales along lateral row; 4.5 scales between dorsal-fin origin and lateral line; barbels absent; caudal blotch small, rounded; humeral blotch present, small, rounded, on 3rd and 4th lateral-line scale	Linthoingambi & Vishwanath (2007); Batuwita et al. (2015); Katwate et al. (2015)
P. yuensis	(Arunkumar & Tombi Singh 2002)	India: Manipur, Maklang River, 21 kilometers from Moreh, Chindwin drainage	Northeast India: Manipur, Chindwin drainage	Lateral line incomplete with 6-9 pored, 21-22 scales along lateral row; 4.5 scales between dorsal-fin origin and lateral line; barbels absent; caudal blotch rounded; humeral blotch present, small, rounded, on 3rd and 4th lateral-line scales	Arunkumar et al. (2002)
***Pethia* sensu lato**					
"P." aurea	Knight 2013	India: West Bengal, Ponds in South 24 – Parganas district	Northeast India: West Bengal, Ponds in South 24 – Parganas district	Lateral line incomplete with 3-4 pored, 25-26 scales along lateral row; 5.5 scales between dorsal-fin origin and lateral line; barbels absent; caudal blotch elongated as a bar; humeral blotch absent; additional blotch beneath the origin of	Knight (2013)

				dorsal fin; black spot present at base of dorsal and anal fins	
"P." canius	(Hamilton 1822)	India: West Bengal, Pond in Cooch, Behar District (neotype designated by Knight, 2013)	Northeast India: West Bengal, Pond in Cooch, Behar District	Lateral line incomplete with 3-4 pored, 20-21 scales along lateral row; 4.5 scales between dorsal-fin origin and lateral line; barbels absent; caudal blotch elongated as a bar; humeral blotch present; additional blotch beneath the origin of dorsal fin; black spot present at base of dorsal, anal and pelvic fins	Knight (2013)
"P." gelius	(Hamilton 1822)	India: West Bengal, Totapara in Jalpaiguri, Torsa river (neotype designated by Knight, 2013)	Northeast India: West Bengal, Totapara in Jalpaiguri, Torsa river	Lateral line incomplete with 3-4 pored, 21-22 scales along lateral row; 4.5 scales between dorsal-fin origin and lateral line; barbels absent; caudal blotch elongated as a bar; humeral blotch present; additional blotch beneath the origin of dorsal fin; black spot present at base of dorsal and anal fins	Knight (2013)
"P." guganio	(Hamilton 1822)	India: Brahmaputra and Yamuna rivers	Northeast India: Ganga-Brahmaputra drainages	Lateral line complete with 28-29 pored scales; 5.5 scales between dorsal-fin origin and lateral line; barbels [?]; caudal blotch rounded; humeral blotch absent	Pethiyagoda et al. (2012); Knight (2013)
"P." narayani	(Hora 1937)	India: Karnataka, Coorg State, Cauvery drainage	South India: Western Ghats, Cauvery drainage	Lateral line complete with 22 scales; 4 scales between dorsal-fin origin and lateral line; barbels absent; three vertical bars on body [?]	Hora (1937)
"P." sharmai	(Menon & Rema Devi 1993)	India: Tamil Nadu, West Annanagar, Mogappair	South India: Tamil Nadu	Lateral line incomplete with 7 pored scales, 40-41 scales along lateral row; 6.5 scales between dorsal-fin origin and lateral line; maxillary barbels present (rudimentary); caudal blotch rounded; humeral blotch absent	Menon & Rema Devi (1993)
"P." castor	Conway, Pinion & Kottelat 2021	Myanmar: Kachin State, Ayeyarwady River, near Shwegu, 24.218 N, 96.823 E	Myanmar: Kachin State, few sites in the middle Ayeyarwady, between Bhamo and Mandalay	Lateral line incomplete with 9-16 pored, 22-23 scales along lateral row; 4.5 scales between dorsal-fin origin and lateral line; barbels absent; caudal blotch and humeral blotch not apparent	Conway et al. (2021)
"P." pollux	Conway, Pinion & Kottelat 2021	Myanmar: Kachin State, Ayeyarwady River, near Shwegu, 24.304 N, 96.811 E	Myanmar: Kachin State, few sites in the middle Ayeyarwady, between Bhamo and Mandalay	Lateral line incomplete with 8-16 pored, 23-24 scales along lateral row; 4.5 scales between dorsal-fin origin and lateral line; barbels absent; caudal blotch and humeral blotch not apparent	Conway et al. (2021)

Table S2. The comparative genetic dataset representative of Smiliogastrinae and outgroups downloaded from GenBank.

Species	*cytb*	*rag1*	Species	*cytb*	*rag1*
Acheilognathus typus (outgroup)	AB239602	EU292688	*Pethia gelius*	AP013336	NA
Barbodes aurotaeniatus	KP712211	MT483497	*Pethia longicauda*	MG199966	NA
Barbodes banksi	KP659422	NA	*Pethia longicauda*	MG199965	NA
Barbodes banksi	MT483245	NA	*Pethia longicauda*	MG199964	NA
Barbodes banksi	MT483244	NA	*Pethia longicauda*	KJ681101	NA
Barbodes binotatus	KP712255	NA	*Pethia longicauda*	KJ681102	NA
Barbodes binotatus	MT483247	MT483499	*Pethia lutea*	KJ681103	NA
Barbodes binotatus	MT483246	MT483498	*Pethia meingangbii*	NA	KJ548126
Barbodes everetti	NA	MT483500	*Pethia melanomaculata*	EU604678	NA
Barbodes lateristriga	MT483249	MT483502	*Pethia narayani*	KP712173	MT483522
Barbodes lateristriga	MT483248	MT483501	*Pethia nigripinna*	MG199967	NA
Barbodes rhombeus	KP659413	MT483504	*Pethia nigrofasciata*	HM536821	KP712482
Barbodes rhombeus	MT483250	MT483503	*Pethia nigrofasciata*	JF793612	NA
Barbodes sp	MT483251	MT483505	*Pethia nigrofasciata*	AY708247	NA
Barboides britzi	EF151089	NA	*Pethia nigrofasciata*	MT483273	MT483523
Barboides gracilis	KP712184	KT199349	*Pethia padamya*	KT159942	NA
Barboides gracilis	NA	KY356484	*Pethia padamya*	KP712225	MT483525
Dawkinsia apsara	MT334786	NA	*Pethia padamya*	MT483274	MT483526
Dawkinsia apsara	MT334785	NA	*Pethia phutunio*	KJ681108	NA
Dawkinsia arulius	MT483253	NA	*Pethia phutunio*	KP659417	MT483527
Dawkinsia arulius	MT483252	NA	*Pethia phutunio*	EU241459	NA
Dawkinsia arulius	MT334787	NA	*Pethia pookodensis*	MG199968	NA
Dawkinsia assimilis	MT483254	NA	*Pethia pookodensis*	MG199969	NA
Dawkinsia assimilis	MT334788	NA	*Pethia punctata*	KJ681107	NA
Dawkinsia austellus	MT334789	NA	*Pethia punctata*	KJ681106	NA
Dawkinsia cf singhala	KP712164	NA	*Pethia punctata*	KJ681104	NA
Dawkinsia crassa	MT334790	NA	*Pethia punctata*	KJ681105	NA
Dawkinsia exclamatio	MT483256	NA	*Pethia punctata*	KM364559	NA
Dawkinsia filamentosa	JQ795445	NA	*Pethia punctata*	KM364557	NA
Dawkinsia filamentosa	JQ795447	NA	*Pethia punctata*	KM364558	NA
Dawkinsia filamentosa	JQ795448	NA	*Pethia reval*	EU604674	NA
Dawkinsia filamentosa	MT483259	NA	*Pethia sanjaymoluri*	MH329834	NA
Dawkinsia filamentosa	MT483258	MT483507	*Pethia sanjaymoluri*	MH329835	NA

Dawkinsia filamentosa	MZ302379	NA	*Pethia sanjaymoluri*	MH329836	NA
Dawkinsia filamentosa	MZ302384	NA	*Pethia sanjaymoluri*	MH329831	NA
Dawkinsia filamentosa	MZ302390	NA	*Pethia sanjaymoluri*	MH329833	NA
Dawkinsia filamentosa	MT732734	MT732769	*Pethia sanjaymoluri*	MH329830	NA
Dawkinsia filamentosa	MZ302389	NA	*Pethia sanjaymoluri*	MH329826	NA
Dawkinsia filamentosa	MZ302386	NA	*Pethia sanjaymoluri*	MH329827	NA
Dawkinsia filamentosa	MZ302387	NA	*Pethia sanjaymoluri*	MH329828	NA
Dawkinsia filamentosa	MT334806	NA	*Pethia sanjaymoluri*	MH329812	NA
Dawkinsia filamentosa	MT334805	NA	*Pethia sanjaymoluri*	MH329814	NA
Dawkinsia filamentosa	MT334801	NA	*Pethia sanjaymoluri*	MH329815	NA
Dawkinsia filamentosa	MT334800	NA	*Pethia sanjaymoluri*	MH329819	NA
Dawkinsia filamentosa	MT334795	NA	*Pethia sanjaymoluri*	MH329820	NA
Dawkinsia filamentosa	MT334794	NA	*Pethia sanjaymoluri*	MH329821	NA
Dawkinsia filamentosa	MT334793	NA	*Pethia sanjaymoluri*	MH329811	NA
Dawkinsia filamentosa	MT334792	NA	*Pethia sanjaymoluri*	KT159940	NA
Dawkinsia filamentosa	EU241455	NA	*Pethia sanjaymoluri*	KT159941	NA
Dawkinsia filamentosa	NA	MT483506	*Pethia sanjaymoluri*	MH329810	NA
Dawkinsia lepida	MT334809	NA	*Pethia sanjaymoluri*	KT159938	NA
Dawkinsia lepida	MT334808	NA	*Pethia sanjaymoluri*	KT159939	NA
Dawkinsia lepida	MT334807	NA	*Pethia setnai*	KF977530	NA
Dawkinsia rohani	MT334814	NA	*Pethia setnai*	KF977529	NA
Dawkinsia rubrotinctus	MT334816	NA	*Pethia setnai*	KF977531	NA
Dawkinsia srilankensis	MT732733	MT732768	*Pethia setnai*	KF977532	NA
Dawkinsia tambraparniei	MT483260	MT483508	*Pethia sp*	MT483288	MT483543
Dawkinsia tambraparniei	MT334823	NA	*Pethia stoliczkana*	MT483275	MT483528
Dawkinsia uttara	MT334819	NA	*Pethia stoliczkana*	KP712228	NA
Dawkinsia uttara	MT334818	NA	*Pethia stoliczkana*	NA	KT199343
Desmopuntius foerschi	MT483262	MT483510	*Pethia ticto*	KP861803	NA
Desmopuntius foerschi	MT483261	MT483509	*Pethia ticto*	KP861804	NA
Desmopuntius gemellus	MT483264	MT483511	*Pethia ticto*	JQ795475	NA
Desmopuntius gemellus	MT483263	NA	*Pethia ticto*	JQ795478	NA
Desmopuntius hexazona	KP659416	KY356498	*Pethia ticto*	EU604679	NA
Desmopuntius johorensis	KP712178	MT483513	*Pethia ticto*	AY708250	NA
Desmopuntius johorensis	MT483265	NA	*Pethia ticto*	MT483276	NA
Desmopuntius pentazona	KP712181	MT483514	*Pethia ticto*	KC696520	NA
Desmopuntius rhomboocellatus	KP659412	MT483515	*Pethia ticto*	AB238969	NA

Desmopuntius rhomboocellatus	MT483266	MT483516	*Pethia ticto*	NA	EU711152
Desmopuntius trifasciatus	MT483268	MT483518	*Puntigrus partipentazona*	KP712182	MT483529
Desmopuntius trifasciatus	MT483267	MT483517	*Puntigrus partipentazona*	MT483277	NA
Enteromius apleurogramma	KX178102	MH616629	*Puntigrus tetrazona*	KC631298	KC631231
Enteromius apleurogramma	KX178103	MH616630	*Puntigrus tetrazona*	EU287909	JX074462
Enteromius apleurogramma_1	KT199303	KP965653	*Puntigrus tetrazona*	MT483278	NA
Enteromius fasciolatus	HM536811	KP712477	*Puntius amphibius*	MT483280	NA
Enteromius jae	KP712205	NA	*Puntius amphibius*	MT483279	MT483530
Enteromius radiatus_2	KT199314	KP965681	*Puntius bimaculatus*	MT483242	NA
Enteromius aff. foutensis sp. 1	MK329229	MK414477	*Puntius bimaculatus*	MT732738	MT732773
Enteromius aff. foutensis sp. 2	MK329235	MK414476	*Puntius brevis*	HM536815	KP712483
Enteromius anema	KP712159	KP965652	*Puntius cauveriensis*	MT483281	NA
Enteromius apleurogramma	KX178098	KX274691	*Puntius chola*	KP712212	KT199344
Enteromius apleurogramma	KX178116	KX274718	*Puntius chola*	MT732736	MT732771
Enteromius apleurogramma	KX178166	KX274725	*Puntius dorsalis*	MT732735	MT732770
Enteromius aspilus	KP659406	KP965654	*Puntius masyai*	HM536817	MT483534
Enteromius atkinsoni	KT199305	KP965655	*Puntius semifasciolatus*	MT483285	MT483536
Enteromius baudoni	KP712198	KP965656	*Puntius semifasciolatus*	MT483239	MT483540
Enteromius brazzai	KP712199	KP965657	*Puntius semifasciolatus*	MT483238	MT483539
Enteromius callipterus	KP712230	KP965658	*Puntius semifasciolatus*	NA	MT483538
Enteromius camptacanthus	KP712231	KP965659	*Puntius semifasciolatus*	NA	MT483535
Enteromius cercops	NA	KX274702	*Puntius snyderi*	MT483241	MT483542
Enteromius cf. guirali	KP712233	KP965660	*Puntius snyderi*	MT483240	MT483541
Enteromius cf. paludinosus	KX178188	KX274708	*Puntius sophore*	MT483287	NA
Enteromius cf. paludinosus	KX178191	KX274709	*Puntius terio*	KP712218	MT483544
Enteromius cf. paludinosus	KX178141	KX274704	*Puntius titteya*	MT732737	MT732772
Enteromius cf. paludinosus	KX178124	KX274719	*Puntius titteya*	NA	MT483545
Enteromius cf. paludinosus	KX178152	KX274724	*Puntius vittatus*	MT732739	MT732774
Enteromius cf. paludinosus	KX178180	KX274698	*Puntius vittatus*	MT732740	MT732775
Enteromius cf. paludinosus "Jipe"	KX178083	KX274715	*Rhynchocypris percnurus* (outgroup)	AP009061	EU409627
Enteromius cf. paludinosus "Jipe"	KX178175	KX274697	*Rohtee ogilbii*	AP011362	KF029704
Enteromius cf. pleurogramma	KP712245	KP965661	*Sahyadria chalakkudiensis*	JX311437	NA
Enteromius eburneensis	KP712201	KP965662	*Sahyadria denisonii*	JQ795471	NA
Enteromius foutensis s. s.	MF135219	MK414478	*Sahyadria denisonii*	JQ795469	NA
Enteromius holotaenia	KP659415	KP965664	*Sahyadria denisonii*	JX470430	NA
Enteromius hulstaerti	KP712202	NA	*Sahyadria denisonii*	JX470431	NA

Enteromius jacksoni	KX178087	KX274700	*Sahyadria denisonii*	KP712180	MT483546
Enteromius kerstenii	KX178113	KX274717	*Sahyadria denisonii*	MT483290	NA
Enteromius kerstenii	KX178136	KX274721	*Sahyadria denisonii*	EU241453	NA
Enteromius kerstenii	KX178137	KX274722	*Sahyadria denisonii*	KF019637	NA
Enteromius kerstenii	KX178168	KX274726	*Sahyadria denisonii*	AP011244	NA
Enteromius kerstenii	KP712168	KP965667	*Striuntius lineatus*	KP712213	MT483547
Enteromius kerstenii	NA	KX274727	*Systomus asoka*	MT732688	MT732741
Enteromius kerstenii_2	KT199307	KP965668	*Systomus martenstyni*	MT732704	MT732752
Enteromius kerstenii_3	KT199308	KP965669	*Systomus martenstyni*	MT732702	MT732751
Enteromius laticeps	KT199309	KP965670	*Systomus orphoides*	MH688217	NA
Enteromius leonensis	JX074245	KP965671	*Systomus orphoides*	KP712214	KT199346
Enteromius macinensis	KP712162	KP965673	*Systomus orphoides*	NA	MT483548
Enteromius macrops	AF180832	KP965674	*Systomus plerotaenia*	MT732693	NA
Enteromius martorelli	KP712169	NA	*Systomus plerotaenia*	MT732697	NA
Enteromius miolepis	KP712170	KP965675	*Systomus plerotaenia*	MT732700	MT732749
Enteromius neumayeri	KX178104	KX274692	*Systomus pleurotaenia*	MT732689	MT732742
Enteromius neumayeri	KX178131	KX274720	*Systomus pleurotaenia*	MT732694	MT732745
Enteromius neumayeri	KX178153	KX274695	*Systomus pleurotaenia*	MT732695	NA
Enteromius nyanzae	KX178091	KX274701	*Systomus pleurotaenia*	MT732699	NA
Enteromius paludinosus	KT199310	KP965676	*Systomus pleurotaenia*	MT732701	MT732750
Enteromius paludinosus	KT199311	KP965677	*Systomus pleurotaenia*	NA	MT732747
Enteromius perince	KP712158	KP965678	*Systomus pleurotaenia*	NA	MT732746
Enteromius prionacanthus	KP712203	KP965680	*Systomus pleurotaenia*	NA	MT732744
Enteromius radiatus	KX178196	KX274713	*Systomus sarana*	KF574722	NA
Enteromius sp.	KX178200	KX274714	*Systomus sarana*	KF574724	NA
Enteromius sp. "Jipe"	KX178171	KX274705	*Systomus sarana*	KF574727	NA
Enteromius sp. "Jipe"	KX178177	KX274706	*Systomus sarana*	KF574731	NA
Enteromius sp. 4	KX178195	KX274712	*Systomus sarana*	KP712217	MT483550
Enteromius sp. Lango	KP712215	KP965682	*Systomus sarana*	KP712210	KT199347
Enteromius taeniurus	KP659407	NA	*Systomus sarana*	JQ795491	NA
Enteromius thysi	KP659408	KP965683	*Systomus sarana*	JQ795489	NA
Enteromius trimaculatus	AB239600	EU711148	*Systomus sarana*	JQ795493	NA
Enteromius trispilopleura	KX178192	KX274710	*Systomus sarana*	KF574728	NA
Enteromius trispilopleura	KX178193	KX274711	*Systomus sarana*	KF574736	NA
Enteromius trispilos	KP712232	KP965685	*Systomus sarana*	KF574737	NA
Enteromius yongei	KX178084	KX274699	*Systomus sarana*	HM010726	MT483549

Gobio gobio (outgroup)	AB239596	EU292689	*Systomus sarana*	MT483291	MT483551
Haludaria fasciata	JQ795450	NA	*Systomus sarana*	MT732711	MT732757
Haludaria fasciata	JQ795452	NA	*Systomus sarana*	MT732712	NA
Haludaria fasciata	JX074247	GQ913442	*Systomus sarana*	MT732713	NA
Haludaria melanampyx	MT483270	NA	*Systomus sarana*	MT732718	NA
Hampala dispar	KC631297	KC631230	*Systomus sarana*	MT732717	NA
Hampala dispar	KP712166	NA	*Systomus sarana*	MT732710	NA
Hampala macrolepidota	MH688236	NA	*Systomus sarana*	MT732707	MT732753
Hampala macrolepidota	JQ346142	JQ346122	*Systomus sarana*	MT732719	NA
Hampala macrolepidota	HM536790	NA	*Systomus sarana*	MT732720	NA
Hampala macrolepidota	NA	EU409623	*Systomus sarana*	MT732721	MT732761
Hampala macrolepidota	NA	KP712480	*Systomus sarana*	MT732722	NA
Oliotius oligolepis	HM536820	KP712481	*Systomus sarana*	MT732723	NA
Opsariichthys uncirostris (outgroup)	AB218897	FJ197126	*Systomus sarana*	MT732725	MT732762
Oreichthys cosuatis	HM536822	GQ913441	*Systomus sarana*	MT720888	NA
Oreichthys parvus	KC631299	KC631233	*Systomus sarana*	MT720889	NA
Osteobrama belangeri	KP712244	NA	*Systomus sarana*	MT720890	NA
Osteobrama cotio	KP712183	KF029702	*Systomus sarana*	MT720891	NA
Osteobrama cunma	KP712235	KF029703	*Systomus sarana*	MT720893	NA
Osteobrama feae	KP712242	NA	*Systomus sarana*	MT720894	NA
Osteobrama vigorsii	KF574712	NA	*Systomus sarana*	MT720895	NA
Osteobrama vigorsii	KF574713	NA	*Systomus sarana*	MT720896	NA
Pethia bandula	JF793597	NA	*Systomus sarana*	MT720897	NA
Pethia bandula	EU604673	NA	*Systomus sarana*	NA	MT732763
Pethia bandula	AY708244	NA	*Systomus sarana*	NA	MT732759
Pethia conchonius	KT159943	NA	*Systomus sarana*	NA	MT732754
Pethia conchonius	KT159945	NA	*Waikhomia hira*	MT090749	NA
Pethia conchonius	KP712226	KT199342	*Waikhomia hira*	MT090748	NA
Pethia conchonius	JF793624	NA	*Waikhomia hira*	MT090747	NA
Pethia conchonius	MT483272	NA	*Waikhomia sahyadriensis*	MT090759	NA
Pethia conchonius	AY004751	NA	*Waikhomia sahyadriensis*	MT090758	NA
Pethia conchonius	JQ795488	NA	*Waikhomia sahyadriensis*	MT090757	NA
Pethia conchonius	JQ795486	NA	*Waikhomia sahyadriensis*	MT090756	NA
Pethia conchonius	MT483271	MT483520	*Waikhomia sahyadriensis*	MT090755	NA
Pethia cumingii	KP712177	KY356497	*Waikhomia sahyadriensis*	MT090754	NA
Pethia cumingii	AY708249	NA	*Waikhomia sahyadriensis*	MT090753	NA

Pethia cumingii	EU604676	NA	*Waikhomia sahyadriensis*	MT090752	NA
Pethia cumingii	EU604675	NA	*Waikhomia sahyadriensis*	MT090751	NA
Pethia cumingii	AP011316	NA	*Waikhomia sahyadriensis*	MT090750	NA
Pethia gelius	JQ795481	NA			
Pethia gelius	JQ795483	NA			
Pethia gelius	EU241456	NA			

Table S3. Nucleotide substitution models and the partitions used in the phylogenetic analyses.

**Analysis**	**Gene**	**Number of sequences**	**Model selector**	**Partition**	**Model**
Bayesian inference: MrBayes	*cytb* (1082 bp)	395	PartitionFinder 2	*cytb* 1st	SYM+I+G
				cytb 2nd	HKY+I+G
				cytb 3rd	GTR+G
	rag1 (1490 bp)	204	PartitionFinder 2	*rag1* 1st + *rag1* 2nd	HKY+I+G
				rag1 3rd	SYM+G
	cytb + *rag1* (2572 bp)	371	PartitionFinder 2	*cytb* 1st	SYM+I+G
				cytb 2nd	HKY+I+G
				cytb 3rd	GTR+G
				rag1 1st + *rag1* 2nd	K80+I+G
				rag1 3rd	SYM+G
Maximum likelihood inference:	*cytb* (1082 bp)	395	ModelTest-NG	*cytb* 1st	GTR+I+G4
				cytb 2nd	GTR+I+G4
				cytb 3rd	GTR+I+G4
	rag1 (1490 bp)	204	ModelTest-NG	*rag1* 1st	GTR+I+G4
				rag1 2nd	GTR+I+G4
				rag1 3rd	GTR+I+G4
	cytb + *rag1* (2572 bp)	371	ModelTest-NG	*cytb* 1st	GTR
				cytb 2nd	HKY
				cytb 3rd	GTR
				rag1 1st	GTR
				rag1 2nd	GTR
				rag1 3rd	GTR
Starting tree for Molecular species delimitation using bPTP, mPTP: IQ-TREE	*cytb* (1082 bp)	145	ModelTest-NG	*cytb*	GTR+I+G4

Table S4. Specimens of *Pethia* examined for the morphological analysis. LK, Sri Lanka; IND, India.

Species	Material
Pethia bandula (LK)	ZRC38483 (CMK7245),1, Holotype, 34.6 mm SL,Kelani basin,Minimaru Coloniya, Galapitamada; CMK7146, Paratypes, 6,28.4-31.6 mm SL,Kelani basin,Minimaru Coloniya, Galapitamada
Pethia cumingii (LK)	DZ3305,1,39.7 mm SL, Bentara basin, Bambarawana; WHT30054,2,38.2-41.7 mm SL, Bentara basin, Bambarawana; WHT30544,1,35.1 mm SL, Bentara basin, Bambarawana; WHT7584,4,38.7-40.5 mm SL, Bentara basin, Bambarawana; DZ4398,1,41.5 mm SL, Gin basin, Homadola, Udugama; DZ4959,1,35.8 mm SL, Gin basin, Hiniduma; DZ4960,1,29.5 mm SL, Gin basin, Hiniduma; DZ4961,1,30.4 mm SL, Gin basin, Hiniduma; DZ4962,1,26.1 mm SL, Gin basin, Hiniduma; DZ4963,1,26.3 mm SL, Gin basin, Hiniduma; DZ5014,3,26.1-27.8 mm SL, Gin basin, Hiniduma; WHT1794,7,33,39.6 mm SL, Gin basin, Neluwa; WHT30070,10,24.6-35.5 mm SL, Gin basin, Neluwa; WHT30339,2,35.5-39.2 mm SL, Gin basin, Homadola, Udugama; WHT30432,2,26.3-26.5 mm SL, Gin basin, Koralegama, Kanneliya; WHT7518,1,36.8 mm SL, Gin basin, Neluwa; DZ3026,1,32.2 mm SL, Kalu basin, Elapatha, Ratnapura; DZ3056,1,28.9 mm SL, Kalu basin, Elapatha, Ratnapura; DZ3917,5,25,34.5 mm SL, Kalu basin, Dombagaskanda; DZ3936,2,27.4-37.1 mm SL, Kalu basin, Elapatha, Ratnapura; DZ4048,1,28.1 mm SL, Kalu basin, Nagahadola; WHT211,2,25.3-27.2 mm SL, Kalu basin, Walandure; WHT30303,5,25.5-32.3 mm SL, Kalu basin, Walandure; WHT404,2,29.1-32.4 mm SL, Kalu basin, Walandure; WHT580,12,28.2-35.1 mm SL, Kalu basin, Dombagaskanda; WHT74,12,23.6-35.5 mm SL, Kalu basin, Dombagaskanda
Pethia reval (LK)	DZ5354,2,30.7-30.9 mm SL, Attanagalu basin, Algama; DZ5365,2,31.3-33.2 mm SL, Attanagalu basin, Morenna; DZ4321,1,22.4 mm SL, Deduru basin, Kolamunu Oya; DZ5000,1,29.0 mm SL, Kelani basin, Yogama; DZ5001,1,32.8 mm SL, Kelani basin, Yogama; DZ5002,1,34.0 mm SL, Kelani basin, Yogama; DZ5003,1,30.4 mm SL, Kelani basin, Yogama; WHT0004,3,27.5-29.4 mm SL, Kelani basin, Labugama; WHT1951,1,33.3 mm SL, Kelani basin, Kitulgala; WHT30111,2,25.3-27.6 mm SL, Kelani basin, Avissawella; WHT30515,1,29.1 mm SL, Kelani basin, Kahahena; WHT677,1,30.4 mm SL, Kelani basin, Labugama; WHT7519,3,28.2-32.6 mm SL, Kelani basin, Avissawella; WHT7536,3,28.8-34.0 mm SL, Kelani basin, Labugama; DZ4828,1,30.1 mm SL, Maha Oya basin, Bopitiya, Giriulla; DZ4829,1,28.5 mm SL, Maha Oya basin, Bopitiya, Giriulla; DZ4830,1,25.8 mm SL, Maha Oya basin, Bopitiya, Giriulla; DZ4831,1,25.9 mm SL, Maha Oya basin, Bopitiya, Giriulla; DZ3278,1,35.0 mm SL, Mahaweli basin, Sarasavi Oya, Peradeniya; DZ4315,3,27.8-30.6 mm SL, Mahaweli basin, Kahawatura Ela; DZ5398,5,30.7-37.1 mm SL, Mahaweli basin, Nillambe
Pethia nigrofasciata (LK)	DZ4452,5,31.4-38.8 mm SL, Attanagalu basin, Alawala; DZ5350,10,29.5-42.9 mm SL, Attanagalu basin, Karasnagoda; DZ5351,12,31.9-40.6 mm SL, Attanagalu basin, Atha-uda kanda; DZ5352,7,30,39.1 mm SL, Attanagalu basin, Udawaka; DZ5353,12,32.1-42.7 mm SL, Attanagalu basin, Algama; DZ5369,1,48.7 mm SL, Attanagalu basin, Algama waterfall; DZ4039,1,42.3 mm SL, Bentara basin, Bambarawana; DZ4059,2,32.3-34.5 mm SL, Bentara basin, Thundola, Horawala; DZ4092,3,42.0-44.5 mm SL, Bentara basin; WHT132,1,32.2 mm SL, Bentara basin, Horawala; WHT30053,2,34.0-36.2 mm SL, Bentara basin, Bambarawana; WHT30322,1,26.7 mm SL, Bentara basin, Beraliya Mukalana; WHT30545,2,39.0-40.1 mm SL, Bentara basin, Bambarawana; DZ4928,1,38.9 mm SL, Gin basin, Kottawa FR; DZ4929,1,33.8 mm SL, Gin basin, Kottawa FR; DZ4930,1,34.3 mm SL, Gin basin, Kottawa FR; DZ4964,1,22.2 mm SL, Gin basin, Hiniduma; DZ4965,1,30.4 mm SL, Gin basin, Hiniduma; WHT10950,4,32.2-35.2 mm SL, Gin basin, Kottawa FR; WHT1788,5,32.0-37.1 mm SL, Gin basin, Neluwa; WHT1846,5,31.2-35.0 mm SL, Gin basin, Mawanana, Neluwa; WHT30071,13,29.7-43.0 mm SL, Gin basin, Mawanana, Neluwa; WHT30665,1,31.3 mm SL, Gin basin, Udugama ela; WHT567,1,28.8 mm SL, Gin basin, Kombala-Kottawa FR; DZ3024,1,39.4 mm SL, Kalu basin, Elapatha, Ratnapura; DZ3025,1,44.7 mm SL, Kalu basin, Elapatha, Ratnapura; DZ3935,2,29.9-37.9 mm SL, Kalu basin, Elapatha, Ratnapura; DZ4037,1,39.8 mm SL, Kalu basin, Athwelthota; DZ4049,5,26.7-45.1 mm SL, Kalu basin, Nagahadola, Niriella; WHT119,3,34.6-39.9 mm SL, Kalu basin, Walandure; WHT226,4,31.5-35.6 mm SL, Kalu basin, Walandure; WHT30102,2,36.2-39.5 mm SL, Kalu basin, Ekneligoda; WHT30389,3,35.8-42.8 mm SL, Kalu basin, Ekneligoda; WHT30587,1,40.8-40.8 mm SL, Kalu basin, Walandure; WHT582,2,27.6-31.6 mm SL, Kalu basin, Athwelthota; WHT7679,4,32.9-38.9 mm SL, Kalu basin, Athwelthota; WHT9203,4,30.2-34.3 mm SL, Kalu basin, Kuruwita; WHT9318,4,33.4-37.3 mm SL, Kalu basin, Kuruwita; DZ4403,7,35.3-43.2 mm SL, Kelani basin, Ilukwatta; WHT0005,13,28.9-42.4 mm SL, Kelani basin, Labugama, Waga; WHT30065,1,32.8 mm SL, Kelani basin, Avissawella; WHT30516,1,23.9 mm SL, Kelani basin, Kahaena; WHT44,2,33.5-34.0 mm SL, Kelani basin, Kitulgala; WHT7677,10,34.0-42.8 mm SL, Kelani basin, Kitulgala; DZ4316,8,21.8-34.0 mm SL, Mahaweli basin, Kahawatura Ela; WHT30215,2,30.9-34.6 mm SL, locality unknown; WHT30694,2,40.0-40.2 mm SL, locality unknown; DZ4509,15,30.0-44.2 mm SL, Nilwala basin, Ugudu dola, Opatha; DZ4834,1,33.3 mm SL, Nilwala basin, Ampanagala; WHT7678,8,28.4-49.2 mm SL, Nilwala basin, Bangama, Akuressa; DZ3454,2,35.6-40.1 mm SL, Walawe basin, Suriyakanda; DZ3911,2,29.5-

	35.4 mm SL, Walawe basin, Suriyakanda; DZ3920,2,41.3-42.8 mm SL, Walawe basin, Pambahinna; DZ4272,13,26.1-39.2 mm SL, Walawe basin, Rajawaka; WHT658,7,29.8-36.9 mm SL, Walawe basin, BelihulOya, pambahinna
Pethia melanomaculata (LK)	WHT30734,1,22.0 mm SL, Deduru? basin, tributary of danduru oya (Deduru?); DZ4660,1,28.4 mm SL, Gal Oya basin, Kotagama; DZ4668,3,29.3-34.8 mm SL, Gal Oya basin, Thummodara, Namal Oya; DZ4885,1,40.6 mm SL, Kala Oya basin, Rajanganaya; DZ4886,1,36.7 mm SL, Kala Oya basin, Rajanganaya; DZ5394,4,37.0-41.5 mm SL, Kala Oya basin, Dambulu Oya, Dambulla; DZ4705,5,29.0-36.2 mm SL, Kumbukkan basin, Bellan Oya, Nakkala; DZ1500,1,32.4 mm SL, Mahaweli basin, Ulhitiya; DZ1501,1,31.2 mm SL, Mahaweli basin, Ulhitiya; DZ3018,1,31.4 mm SL, Mahaweli basin, Ulhitiya; DZ3019,1,29.9 mm SL, Mahaweli basin, Ulhitiya; DZ3020,1,31.8 mm SL, Mahaweli basin, Ulhitiya; DZ4303,1,27.0 mm SL, Mahaweli basin, Angammedilla, Polonnaruwa; DZ4304,1,30.2 mm SL, Mahaweli basin, Angammedilla, Polonnaruwa; DZ4305,1,31.2 mm SL, Mahaweli basin, Angammedilla, Polonnaruwa; FF728A,1,35.5 mm SL, Mahaweli basin, Kantale Tank; FF728B,3,29.1-30.2 mm SL, Mahaweli basin, Kantale Tank; WHT1861,4,25.1-37.8 mm SL, Mahaweli basin, Hasalaka; WHT218,2,30.2-31.4 mm SL, Mahaweli basin, Habarana; WHT7719,2,32.1-34.1 mm SL, Mahaweli basin, Hasalaka; WHT7748,9,30.9-37.2 mm SL, Mahaweli basin, Hasalaka; DZ4104,1,34.1 mm SL, Malwathu basin, Polonnaruwa; DZ5255,11,23.4-31.8 mm SL, Malwathu basin, Thulawelliya, Medawacchiya; WHT30280,3,29.1-30.8 mm SL, Malwathu basin, Anuradhapura; WHT30184,3,36.3-43.0 mm SL, locality unknown
Pethia ticto (IND)	WHT7720,1,33.6 mm SL, Boncron, West Bengal; WHT7718,5,30.8-34.7 mm SL, Nadia, West Bengal
Pethia cf. *ticto* (IND)	WHT30122,2,31.8-33.0 mm SL, Netravadi River
Pethia cf. *punctata* (IND)	WHT318,2,40.8-45.9 mm SL, 1 km south of Pathanamitha, Kerala; WHT30404,1,37.3-37.3 mm SL, Kalimanoor; WHT339,1,38.4-38.4 mm SL, Kumarakom, Kotteyam; WHT30090,1,40.0-40.0 mm SL, Kuthyaparumba; WHT30122,1,32.8 mm SL, Netravadi R; WHT7717,7,34.4-37.2 mm SL, Netravadi R, 1 km downstream of Uppinangadi; WHT7747,9,32.2-37.3 mm SL, Netravadi R, 1 km downstream of Uppinangadi; WHT7716,3,33.7-37.6 mm SL, South of Calicut, Thrissur road; WHT7715,3,30.4-33.7 mm SL, Thenmalai Dam; WHT359,2,44.5-44.9 mm SL, Thrissur, Peechi Dam
Pethia conchonius (IND)	WHT10940,5,53.6-56.0 mm SL, Nadia, West Bengal
Pethia cf. *conchonius* (IND)	WHT30367,1,55 mm SL, Madikari, Karnataka; WHT30079,3,44.6-46.3 mm SL, Srirangapatana, Cauvery River

Table S5. Intraspecific uncorrected pairwise *cytb* genetic distances for species of *Pethia* in Sri Lanka.

cytb **(%)**	**[1]**	**[2]**	**[3]**	**[4]**	**[5]**	**[6]**	**[7]**	**[8]**
[1] *P. nigrofasciata* (subclade 1)	0.0-2.4							
[2] *P. nigrofasciata* (subclade 2)	2.2-4.0	0.0-0.4						
[3] *P. bandula*	0.8-2.0	3.0-3.2	0.0					
[4] *P. reval* (subclade A)	4.6-6.4	5.2-6.0	4.6-5.2	0.0-1.0				
[5] *P. reval* (subclade B)	5.2-6.6	5.6-6.0	5.6-5.8	1.8-2.2	0.0-0.6			
[6] *P. cumingii* (subclade C)	4.4-6.0	5.0-5.6	4.8-5.2	1.2-2.2	1.6-2.6	0.0-0.8		
[7] *P. cumingii* (subclade D)	4.4-5.6	5.6-5.8	4.8	1.0-1.6	1.8-2.0	1.2-1.8	0.0	
[8] *Pethia melanomaculata*	7.4-8.4	7.6-8.4	7.8-8.2	6.2-7.2	6.4-7.0	6.8-7.6	7.0-7.4	0.0-0.6

Table S6. Genetic diversity, based on *cytb* and *rag1*, in Sri Lankan species of *Pethia*. Number of sequences (N), number of haplotypes (h), polymorphic sites (S), parsimony-informative sites (P), nucleotide diversity (π), haplotype diversity (Hd). None of the neutrality tests was statistically significant.

cytb<br>***rag1***	**N**	**h**	**S**	**P**	***π***	**Hd**	**Tajima's D test**	**Fu and Li's F test**
Pethia melanomaculata	12	7	9	7	0.00287	0.864	0.17135	0.50750
	8	2	2	0	0.00034	0.250	-1.31009	-1.51361
Pethia cumingii	15	9	20	16	0.00691	0.914	0.87622	0.78798
	9	4	3	1	0.00057	0.583	-0.93613	-0.85966
Pethia reval	33	12	33	28	0.01078	0.877	1.36098	1.12476
	13	1	0	0	0.0	0.0	NA	NA
Pethia nigrofasciata	39	23	64	49	0.01163	0.970	-0.66449	-0.20228
	18	4	3	2	0.00047	0.595	-0.59106	-0.25383
Pethia cumingii + Pethia reval	48	20	44	37	0.01220	0.927	0.99891	0.90751
	22	4	3	1	0.00024	0.260	-1.47087	-1.56017

Table S7. Component loadings in the principal component analysis of the size-adjusted morphometric measurements of species of *Pethia* in Sri Lanka.

Parameter	All five Sri Lankan species of *Pethia*					*Pethia bandula*, *P. nigrofasciata*, *P.* cf. *nigrofasciata* (Attanagalu)					*Pethia reval*, *P. cumingii*, *P.* cf. *cumingii* (Kalu)				
	**PC1**	**PC2**	**PC3**	**PC4**	**PC5**	**PC1**	**PC2**	**PC3**	**PC4**	**PC5**	**PC1**	**PC2**	**PC3**	**PC4**	**PC5**
Eigenvalue	5.8	1.7	1.5	1.3	1.1	4.8	2	1.8	1.4	1.1	3.4	2.2	1.7	1.5	1.3
% of variance	31.9	9.6	8.2	7.4	6.3	26.5	11.2	10.1	7.8	6.3	19	12	9.4	8.3	7.4
**Component loadings**															
Predorsal length	0.272	0.194	0.022	0.229	-0.095	0.308	-0.247	0.104	0.039	0.04	0.29	-0.041	0.156	0.275	0.209
Postdorsal length	0.121	-0.384	0.16	0.477	0.095	0.018	0.123	0.558	-0.047	0.035	-0.084	0.333	0.097	-0.13	0.334
Preanal length	0.24	0.35	-0.227	0.14	-0.324	0.287	-0.339	-0.025	0.03	0.08	0.311	-0.323	-0.113	0.22	-0.24
Prepelvic length	0.217	0.46	0.019	-0.078	-0.269	0.229	-0.33	-0.285	-0.051	0.277	0.282	-0.286	-0.148	0.35	-0.21
Caudal-peduncle length	-0.13	0.17	0.15	0.456	0.474	-0.19	-0.292	0.264	0.334	0.074	-0.076	-0.228	0.426	0.042	0.261
Caudal-peduncle depth	0.225	-0.242	0.096	0.333	-0.225	0.24	0.026	0.434	-0.005	0.129	0.08	0.222	0.182	0.525	0.109
Body depth	0.324	-0.094	0.019	0.303	-0.181	0.311	-0.157	0.317	-0.041	0.153	0.234	0.192	0.281	0.223	0.256
Dorsal-fin height	0.223	-0.095	-0.432	-0.024	0.141	0.203	0.168	-0.098	0.502	0.18	0.317	-0.007	0.291	-0.216	0.095
Dorsal-fin base length	0.271	-0.331	0.201	-0.215	-0.037	0.207	0.452	-0.003	-0.293	0.1	0.093	0.484	-0.177	-0.103	-0.068
Anal-fin height	0.165	0.014	-0.397	-0.089	0.487	0.173	0.199	-0.062	0.5	-0.318	0.159	-0.305	0.234	-0.234	0.105
Anal-fin base length	0.132	-0.288	0.293	-0.393	-0.103	0.168	0.355	-0.168	-0.175	0.444	0.093	0.393	0.126	0.322	-0.307
Pelvic-fin length	0.285	-0.198	-0.184	-0.164	0.138	0.275	0.282	-0.072	0.324	0.012	0.212	0.243	0.253	-0.217	-0.348
Pectoral-fin length	0.265	-0.033	-0.076	-0.062	0.218	0.236	0.074	-0.01	0.173	0.147	0.254	0.033	0.348	-0.26	-0.279
Head length	0.233	0.299	0.358	-0.091	0.281	0.298	-0.243	-0.123	-0.037	-0.131	0.351	0.011	-0.208	-0.064	0.275
Head depth	0.284	0.069	0.099	-0.093	-0.044	0.256	-0.013	-0.109	-0.295	-0.055	0.205	0.033	-0.1	-0.129	-0.305
Snout length	0.238	0.212	0.351	-0.139	0.271	0.187	-0.149	-0.314	-0.053	-0.256	0.238	0.094	-0.352	0.008	0.265
Eye diameter	0.181	-0.048	-0.336	-0.02	-0.026	0.18	0.157	0.058	-0.118	-0.601	0.282	-0.047	0.008	-0.167	0.122
Interorbital width	0.303	-0.008	0.079	0.09	0.077	0.29	-0.025	0.248	-0.144	-0.24	0.336	0.091	-0.297	-0.172	0.16

Table S8. Proportional morphometric data for the species of *Pethia* in Sri Lanka.

	Pethia melanomaculata (n = 49)				*P. reval* (n = 35)				*P. cumingii* (n = 80)				*P. nigrofasciata* (n = 215)				*P. bandula* (n = 7)			
	**min**	**max**	**mean**	**sd**	**min**	**max**	**mean**	**sd**	**min**	**max**	**mean**	**sd**	**min**	**max**	**mean**	**sd**	**min**	**max**	**mean**	**sd**
Standard length (mm)	23.4	41.5			22.4	37.1			23.6	41.7			21.8	49.2			28.4	34.6		
**In percent of standard length**																				
Predorsal length	51.4	56.4	54.4	1.1	52.3	57.5	55.3	1.1	52.7	65.3	55.8	1.6	51.7	62.0	56.4	1.5	55.5	58.5	56.9	1.1
Postdorsal length	49.0	56.6	54.1	1.6	52.2	57.6	55.3	1.4	51.2	58.4	55.6	1.3	50.6	59.4	55.8	1.6	54.8	57.8	56.3	1.2
Preanal length	66.8	73.0	70.4	1.6	68.8	75.9	72.0	1.6	68.4	76.6	72.2	1.8	66.8	77.6	72.3	1.6	70.1	74.3	72.9	1.5
Prepelvic length	46.8	51.9	49.0	1.3	47.9	54.1	50.1	1.5	46.5	53.0	49.8	1.3	46.5	55.0.	50.7	1.3	49.3	51.8	50.1	0.9
Caudal-peduncle length	17.4	23.9	20.1	1.4	15.5	21.6	19.1	1.5	14.9	21.0	18.4	1.3	13.5	25.3	19.2	1.9	17.0	19.3	18.4	1.0
Caudal-peduncle depth	11.7	17.0	14.5	1.1	14.3	16.6	15.4	0.6	12.5	16.8	15.1	0.8	12.6	18.0.	15.5	0.8	15.8	16.5	16.1	0.3
Body depth	32.3	42.2	38.0	2.7	38.7	45.6	42.0	1.5	38.6	46.4	42.8	1.7	37.2	50.5	44.0	2.4	42.2	48.0	44.5	2.2
Dorsal-fin height	23.9	29.8	26.3	1.5	25.6	31.9	28.5	1.7	25.6	34.9	29.9	1.8	19.6	33.9	27.9	2.2	23.9	27.8	26.0	1.5
Dorsal-fin base length	15.6	21.9	18.3	1.3	17.6	23.5	20.5	1.4	18.2	24.7	20.8	1.5	13.1	27.0	22.2	2.0.	21.4	25.0	23.3	1.2
Anal-fin height	15.4	21.6	17.8	1.5	16.5	21.4	19.1	1.1	16.2	22.3	18.9	1.4	13.7	23.1	18.2	1.8	16.1	20.4	18.5	1.5
Anal-fin base length	10.7	17.1	13.2	1.3	11.1	14.9	13.4	0.8	10.2	16.2	12.9	1.2	9.8	17.6	13.7	1.3	13.0	14.1	13.6	0.4
Pelvic fin length	19.7	24.8	22.5	1.1	21.3	26.0	23.7	1.2	21.9	27.9	24.8	1.3	20.3	28.4	24.5	1.6	22.5	25.7	23.8	1.3
Pectoral-fin length	18.2	25.2	22.0	1.4	19.7	26.1	23.6	1.7	20.7	27.8	24.3	1.4	19.1	28.8	24.3	1.5	24.0.	27.1	25.5	1.1
Head length	24.1	31.0	27.2	1.4	24.3	29.5	26.9	1.3	24.5	31.8	27.4	10	25.5	30.7	28.1	0.9	27.3	29.6	28.6	0.8
Head depth	19.5	23.9	21.9	0.9	21.1	25.2	23.4	1.1	21.4	26.9	23.7	1.1	21.1	26.6	23.6	1.0	23.1	26.1	24.6	0.9
**In percent of head length**																				
Snout length	23.9	31.7	28.0	2.1	24.1	36.5	28.7	2.6	21.0	39.4	30.1	3.1	26.3	39.4	32.5	2.3	28.7	33.7	30.8	1.9
Eye diameter	30.0	38.3	34.6	1.9	34.4	42.9	38.1	2.3	30.9	47.3	39.0.	3.1	26.2	41.0.	34.1	2.9	29.9	36.9	33.9	2.7
Interorbital width	32.9	45.5	39.1	2.5	36.7	48.2	41.5	2.4	36.9	47.7	42.7	2.4	34.2	46.5	42.0	2.2	42.4	45.5	44.0	1.2

Table S9. Frequency distribution of selected meristic data in the Sri Lankan species of *Pethia* examined in the present study.

**Species**		**Lateral line**		**Pored lateral-line scales**																		
	**N**	**complete**	**incomplete**	**3**	**4**	**5**	**6**	**7**	**8**	**9**	**10**	**11**	**12**	**13**	**15**	**16**	**17**	**18**	**19**	**20**	**21**	**22**
Pethia melanomaculata	52	5	47			2	4	7	5	5	6	5	4	3	5	1		1	2		2	
P. reval	32	0	32		3	8	15	6														
P. cf. *cumingii* (Kalu)	41		41	1	2	9	14	13	1	1												
P. cumingii	34		34		1	14	11	7	1													
P. nigrofasciata	167	166	1						1								1	9	62	64	23	7
P. cf. *nigrofasciata* (Attanagalu)	47	17	30				5	2	6	3	2	4	3	2	1	2			6	9	2	
P. bandula	7	0	7					2	1	3	1											
		**Scales between dorsal-fin origin and lateral line**					**Scales along lateral row**						**Scales between lateral line and pelvic-fin origin**				**Scales between lateral line and anal-fin origin**					
	**N**	**3**	**3.5**	**4**	**4.5**	**5.5**	**17**	**18**	**19**	**20**	**21**	**22**	**2.5**	**3**	**3.5**	**4**	**2**	**2.5**	**3**	**3.5**	**4**	
Pethia melanomaculata	52			3	49			3	9	24	11	7	3	39	10		1	1	49	1		
P. reval	32		31		1		1	10	15	5	1			23	9			2	29	1		
P. cf. *cumingii* (Kalu)	41	1	40				2	18	14	7				36	5			2	37	2		
P. cumingii	34	5	28		1		2	19	9	4				31	3				32	2		
P. nigrofasciata	167			2	164	1	1	9	63	64	23	7		57	109	1			150	13	4	
P. cf. *nigrofasciata* (Attanagalu)	47			2	45				18	22	6	1		18	29			1	43	3		
P. bandula	7				7				1	2	4			5	2			3	4			

Table S10. Proportional morphometric data for *Pethia bandula*, *P. nigrofasciata* (excluding Attanagalu populations), and *P.* cf. *nigrofasciata* (Attanagalu population).

	P. bandula (n = 7)				*P.* cf. *nigrofasciata* (Attanagalu) (n = 47)				*P. nigrofasciata* (n = 168)			
	**min**	**max**	**mean**	**sd**	**min**	**max**	**mean**	**sd**	**min**	**max**	**mean**	**sd**
Standard length (mm)	28.4	34.6			29.5	48.7			21.8	49.2		
**In percent of standard length**												
Predorsal length	55.5	58.5	56.9	1.1	53.8	59.4	56.6	1.5	51.7	62.0	56.4	1.5
Postdorsal length	54.8	57.8	56.3	1.2	50.6	57.1	54.8	1.3	52.4	59.4	56.1	1.5
Preanal length	70.1	74.3	72.9	1.5	69.6	75.7	72.5	1.3	66.8	77.6	72.3	1.7
Prepelvic length	49.3	51.8	50.1	0.9	48.0	55.0	51.3	1.3	47.0	54.1	50.5	1.3
Caudal peduncle length	17.0	19.3	18.4	1.0	14.4	21.9	17.7	1.6	13.5	25.3	19.6	1.8
Caudal peduncle depth	15.8	16.5	16.1	0.3	14.0	17.6	15.3	0.8	12.6	18.0	15.5	0.8
Body depth	42.2	48.0	44.5	2.2	40.7	49.3	44.1	1.8	37.2	50.5	43.9	2.5
Dorsal-fin height	23.9	27.8	26.0	1.5	24.5	31.9	28.3	1.7	19.6	33.9	27.7	2.3
Dorsal-fin base length	21.4	25.0	23.3	1.2	20.9	27.0	23.5	1.7	13.1	25.7	21.9	2.0
Anal-fin height	16.1	20.4	18.5	1.5	14.4	22.0	18.5	1.5	13.7	23.1	18.2	1.9
Anal-fin base length	13.0	14.1	13.6	0.4	13.3	17.6	14.8	1.1	9.8	16.4	13.4	1.2
Pelvic fin length	22.5	25.7	23.8	1.3	21.4	27.6	24.9	1.2	20.3	28.4	24.4	1.7
Pectoral fin length	24.0	27.1	25.5	1.1	20.5	27.5	24.8	1.4	19.1	28.8	24.2	1.5
Head length	27.3	29.6	28.6	0.8	26.4	30.1	28.3	0.9	25.5	30.7	28.0	0.9
Head depth	23.1	26.1	24.6	0.9	21.1	25.7	23.7	1.0	21.2	26.6	23.5	1.0
**In percent of head length**												2.3
Snout length	28.7	33.7	30.8	1.9	28.2	39.4	33.5	2.1	26.3	37.2	32.2	2.9
Eye diameter	29.9	36.9	33.9	2.7	26.2	40.0	33.0	2.4	28.2	41.0	34.4	2.2
Interorbital width	42.4	45.5	44.0	1.2	36.5	44.9	41.6	1.9	34.2	46.5	42.1	1.5

Table S11. Proportional morphometric data for *Pethia reval*, *P. cumingii* (Bentara and Gin populations), and *P.* cf. *cumingii* (Kalu population) in Sri Lanka.

	P. reval (n = 35)				*P.* cf. *cumingii* (Kalu) (n = 41)				*P. cumingii* (n = 39)			
	**min**	**max**	**mean**	**sd**	**min**	**max**	**mean**	**sd**	**min**	**max**	**mean**	**sd**
Standard length (mm)	22.4	37.1			23.6	37.1			24.6	41.7		
**In percent of standard length**												
Predorsal length	52.3	57.5	55.3	1.1	52.7	65.3	55.6	2.0	54.2	59.0	56.0	1.2
Postdorsal length	52.2	57.6	55.3	1.4	51.2	58.4	56.0	1.3	52.7	57.0	55.2	1.0
Preanal length	68.8	75.9	72.0	1.6	68.4	74.2	71.1	1.3	69.3	76.6	73.4	1.6
Prepelvic length	47.9	54.1	50.1	1.5	46.5	52.0	49.1	1.2	48.2	53.0.	50.5	1.1
Caudal-peduncle length	15.5	21.6	19.1	1.5	15.6	21.0	18.6	1.1	14.9	20.9	18.1	1.5
Caudal-peduncle depth	14.3	16.6	15.4	0.6	13.3	16.5	15.0	0.7	12.5	16.8	15.2	0.8
Body depth	38.7	45.6	42.0	1.5	38.6	46.4	42.1	1.8	41.1	46.3	43.5	1.3
Dorsal-fin height	25.6	31.9	28.5	1.7	25.6	32.7	29.7	1.8	27.0	34.9	30.1	1.8
Dorsal-fin base length	17.6	23.5	20.5	1.4	18.2	24.7	20.8	1.5	18.3	23.4	20.9	1.5
Anal-fin height	16.5	21.4	19.1	1.1	16.6	22.3	19.0	1.5	16.2	21.0	18.8	1.3
Anal-fin base length	11.1	14.9	13.4	0.8	10.2	14.9	12.8	1.0	10.2	16.2	13.1	1.3
Pelvic-fin length	21.3	26.0	23.7	1.2	21.9	26.7	24.7	1.2	22.8	27.9	25.0	1.3
Pectoral-fin length	19.7	26.1	23.6	1.7	20.8	27.8	24.3	1.4	20.7	27.3	24.3	1.5
Head length	24.3	29.5	26.9	1.3	25.5	31.8	27.6	1.0	24.5	29.5	27.2	1.0
Head depth	21.1	25.2	23.4	1.1	21.4	26.2	23.8	1.1	21.6	26.9	23.5	1.1
**In percent of head length**												
Snout length	24.1	36.5	28.7	2.6	21.0	35.4	29.3	2.6	22.1	39.4	31.1	3.3
Eye diameter	34.4	42.9	38.1	2.3	32.9	47.3	39.3	3.1	30.9	45.9	38.6	3.0
Interorbital width	36.7	48.2	41.5	2.4	36.9	45.2	41.5	1.9	38.7	47.7	43.8	2.2
